# Oral Administration of *Lactobacillus acidophilus* Expressing GLP-1 Peptide Variants with *N*-Terminal Extensions in Mice with Streptozotocin-Induced Hyperglycemia

**DOI:** 10.3390/ijms272110003

**Published:** 2026-11-08

**Authors:** Arthur A. Lee, Maria B. Chernysheva, Dmitry A. Kashirskikh, Evgenii S. Ruchko, Olesya O. Klychkova, Victoria A. Khotina, Tatiana V. Noskova, Vagif A. Gasanov, Ekaterina A. Vorotelyak, Andrey V. Vasiliev

**Affiliations:** Koltzov Institute of Developmental Biology of the Russian Academy of Sciences, 119334 Moscow, Russia; aal1999arth@gmail.com (A.A.L.); m.b.chern@gmail.com (M.B.C.); dim.kashirsckih@gmail.com (D.A.K.); ruchko7@gmail.com (E.S.R.); olesya_k8@mail.ru (O.O.K.); t.noskova@idbras.ru (T.V.N.); gasanovvagif@gmail.com (V.A.G.); vorotelyak@yandex.ru (E.A.V.); a.v.vasilev@idbras.ru (A.V.V.)

**Keywords:** GLP-1, *Lactobacillus acidophilus*, engineered bacteria, streptozotocin-induced hyperglycemia, glucose tolerance, pancreatic islets

## Abstract

Engineered bacteria are being explored as oral delivery systems for therapeutic peptides. This study examined *Lactobacillus acidophilus* expressing two GLP-1 peptide variants with *N*-terminal extensions, M-GLP-1 and M-6HIS-GLP-1, in male C57BL/6J mice with moderate streptozotocin (STZ)-induced hyperglycemia. Both purified peptides increased insulin concentrations in MIN6 conditioned medium after 24 h, but neither significantly attenuated the STZ-associated reduction in metabolic activity. Cage-level analysis showed lower average post-treatment glucose concentrations in mice receiving *L. acidophilus* expressing M-GLP-1 (Lacto-M-GLP-1) or M-6HIS-GLP-1 (Lacto-M-6HIS-GLP-1) than in STZ controls, but neither group differed significantly from mice receiving empty-vector *L. acidophilus*. The intraperitoneal glucose tolerance test showed numerically lower late-phase glucose concentrations and incremental area under the curve in both groups than in controls. Mice receiving Lacto-M-6HIS-GLP-1 showed greater body-weight change than those receiving Lacto-M-GLP-1 or empty-vector *L. acidophilus*. Pancreatic histology showed a lower pancreatic glucagon-positive signal area and a higher insulin-to-glucagon signal-area ratio than in STZ controls and Lacto-M-GLP-1. The findings demonstrate cellular responses to purified GLP-1 variants and metabolic changes associated with administering *L. acidophilus* expressing the peptides.

## 1. Introduction

Diabetes mellitus comprises a heterogeneous group of metabolic disorders characterized by chronic disruption of glucose homeostasis resulting from impaired insulin secretion, reduced insulin action, or both [1,2]. Dysfunction of pancreatic endocrine cells, including loss or functional impairment of insulin-producing β-cells and inappropriate glucagon secretion by α-cells, contributes substantially to diabetic hyperglycemia [3,4]. Experimental models that reproduce defined disturbances in pancreatic endocrine function therefore remain important for evaluating interventions aimed at improving glucose regulation.

Streptozotocin (STZ) is widely used to induce pancreatic β-cell injury and insulin-deficient hyperglycemia in rodents [5,6]. The severity of the resulting phenotype depends on species, dose, administration schedule, and criteria used to define diabetes. Although STZ-based models do not reproduce the full metabolic complexity of obesity-associated, insulin-resistant type 2 diabetes (T2D), they provide a controlled experimental setting in which glucose regulation can be studied in the presence of pancreatic β-cell injury [5,6].

Glucagon-like peptide-1 (GLP-1) is an important regulator of glucose homeostasis, and the GLP-1 receptor (GLP-1R) is an established therapeutic target [7]. Activation of the GLP-1R enhances glucose-dependent insulin secretion, suppresses inappropriate glucagon secretion, delays gastric emptying, and influences several additional components of metabolic regulation [3,4,8,9,10]. Native GLP-1 is rapidly degraded in vivo, whereas clinically used GLP-1R agonists incorporate structural modifications that increase stability and prolong pharmacological activity [8,10]. These properties, together with the continued reliance on parenteral administration for many GLP-1-based therapies, have stimulated interest in alternative approaches to peptide delivery.

The *N*-terminal region of GLP-1 is particularly important for receptor activation. Structural studies have shown that the *N*-terminus of GLP-1 engages the transmembrane core of GLP-1R and contributes directly to stabilization of the active receptor conformation [11,12,13,14]. Modification of this region can therefore alter receptor binding and signaling. However, selected *N*-terminal substitutions and molecular modifications can preserve GLP-1R agonism despite changes in pharmacological potency [14,15,16]. This is particularly relevant to recombinant peptide production because an initiating methionine or an affinity tag can generate a product with an *N*-terminal extension whose biological activity cannot be inferred directly from that of native GLP-1.

Engineered microorganisms provide a potential approach for oral production and delivery of therapeutic proteins and peptides [17,18]. Lactobacilli have been explored as oral expression and delivery platforms because of their compatibility with gastrointestinal administration, interactions with the intestinal environment, and availability of genetic systems for recombinant expression [19,20]. Depending on construct design, recombinant products may be secreted, displayed at the bacterial surface, or retained intracellularly. For intracellular products, biological availability requires release from bacterial cells and preservation of peptide integrity within the gastrointestinal environment [17,18]. Lactobacilli may also influence host metabolism independently of recombinant peptide production through effects involving intestinal barrier function, host signaling, and interactions with the gut microbial environment [19,21,22].

Previous studies support the feasibility of bacterial delivery of GLP-1-derived peptides. Microbially produced GLP-1 has been shown to improve glucose tolerance through GLP-1R-dependent mechanisms in mice [23], while engineered commensal bacteria producing GLP-1(1–37) induced glucose-responsive insulin production in intestinal epithelial cells [24]. Recombinant lactobacilli producing GLP-1 peptides have also been associated with improved glucose regulation in several experimental models [25,26,27,28]. At the same time, metabolic effects of the bacterial carrier itself have been reported in some studies [29,30], indicating that effects associated with recombinant peptide expression should be interpreted in the context of the corresponding bacterial control.

The present study evaluated two GLP-1 peptide variants with *N*-terminal extensions and *L. acidophilus* expressing these peptides. M-GLP-1 contained an additional initiating methionine preceding native GLP-1(7–37), whereas M-6HIS-GLP-1 additionally contained an *N*-terminal hexahistidine extension. Structural modeling was used to examine the predicted conformations of both variants in complex with GLP-1R. Purified M-GLP-1 and M-6HIS-GLP-1 were evaluated in MIN6 cells for their effects on insulin concentrations in conditioned medium and metabolic activity under high-glucose and STZ exposure conditions. In parallel, *L. acidophilus* expressing M-GLP-1 (Lacto-M-GLP-1) or M-6HIS-GLP-1 (Lacto-M-6HIS-GLP-1) was administered to mice with moderate STZ-induced hyperglycemia, with *L. acidophilus* carrying the empty vector included as a bacterial control. Longitudinal blood glucose concentrations, glucose tolerance, body-weight change, and pancreatic histology were assessed to characterize the metabolic and pancreatic responses associated with these treatments.

## 2. Results

### 2.1. AlphaFold 3 Predicts Similar Peptide Conformations with Different GLP-1R Interface Areas

To assess whether the *N*-terminal extensions were compatible with a receptor-bound conformation similar to that of native GLP-1, complexes of native GLP-1(7–37), M-GLP-1, and M-6HIS-GLP-1 with GLP-1R were modeled using AlphaFold 3. Five predictions were generated for each peptide–receptor complex.

**Figure 1:**
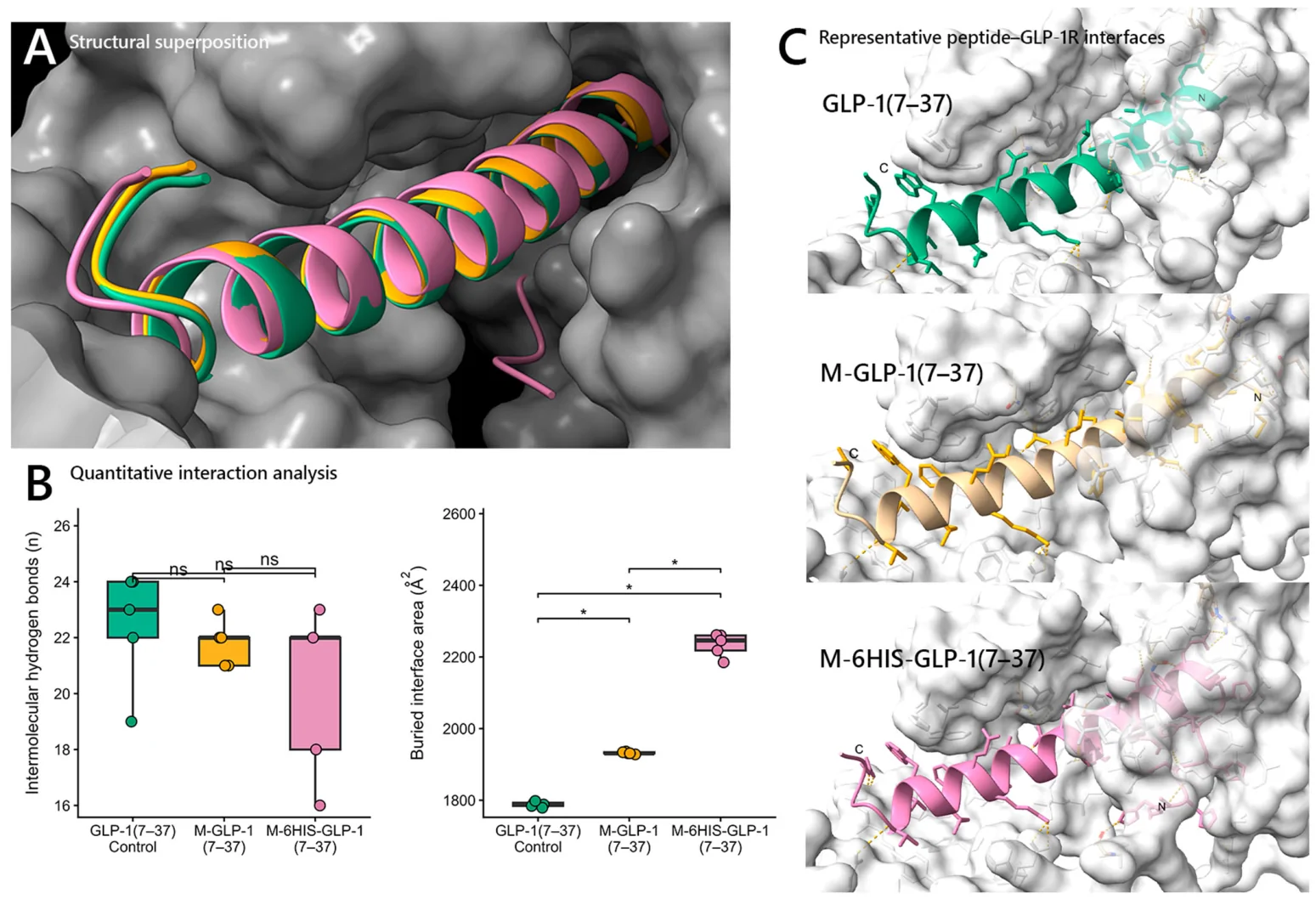
**Structural modeling and comparative analysis of GLP-1(7–37), M-GLP-1, and M-6HIS-GLP-1 in complex with GLP-1R.** (**A**) Structural superposition of representative peptide-GLP-1R complexes after alignment of the GLP-1R structures. Native GLP-1(7–37), M-GLP-1, and M-6HIS-GLP-1 are shown in green, orange, and pink, respectively. (**B**) Comparison of predicted peptide–receptor interface metrics across five AlphaFold 3 predictions for each peptide variant. Intermolecular hydrogen bonds and buried solvent-accessible interface area were calculated in UCSF ChimeraX. Each point represents one predicted complex; box plots show the median and interquartile range. Comparisons were performed using the Kruskal–Wallis test followed by pairwise Wilcoxon rank-sum tests with Holm adjustment for multiple comparisons. * *p* < 0.05; ns, not significant. (**C**) Representative predicted peptide–GLP-1R interfaces showing the receptor surface, peptide conformation, interface residues, and intermolecular hydrogen bonds. The *N*- and *C*-termini of each peptide are indicated. Representative structures were selected based on proximity to the median interface characteristics, with model confidence metrics also taken into account.

The mean buried peptide–receptor interface area across the five predictions was 1788.4 Å^2^ for native GLP-1(7–37), 1932.0 Å^2^ for M-GLP-1, and 2234.0 Å^2^ for M-6HIS-GLP-1. Both recombinant variants showed larger predicted interface areas than native GLP-1, with the largest area observed for M-6HIS-GLP-1. Exploratory pairwise comparisons of the model-derived interface areas showed statistically significant differences among all three peptide variants (Figure 1B).

The corresponding mean numbers of predicted intermolecular hydrogen bonds were 22.4 for native GLP-1, 21.8 for M-GLP-1, and 20.2 for M-6HIS-GLP-1. The higher predicted buried interface areas of the recombinant variants were therefore not accompanied by a greater number of predicted peptide–receptor hydrogen bonds. No statistically significant pairwise differences were detected for this parameter (Figure 1B). Thus, the larger predicted interface areas of the recombinant variants were not accompanied by greater numbers of predicted peptide–receptor hydrogen bonds.

Structural superposition showed broadly similar predicted conformations in complex with GLP-1R, particularly within the central helical region, whereas greater variability was observed toward the peptide termini (Figure 1A,C).

### 2.2. Recombinant GLP-1 Variants Increase Insulin Concentrations in MIN6 Conditioned Medium

**Figure 2:**
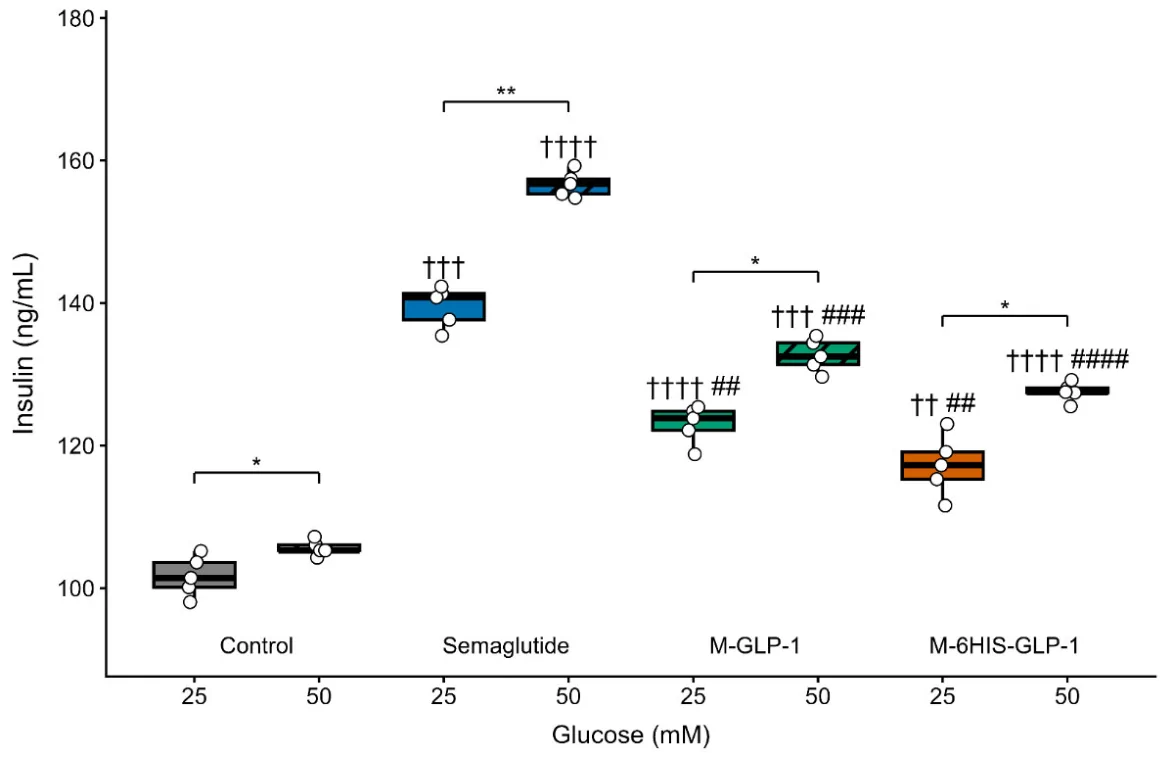
**Effects of recombinant M-GLP-1 and M-6HIS-GLP-1 on insulin concentrations in MIN6 conditioned medium after 24 h.** Each point represents the mean of three technical replicate wells from one independent experiment (*n* = 5 independent experiments per condition). Box plots show the median and interquartile range; whiskers extend to 1.5 × the interquartile range. Data were analyzed by two-way repeated-measures ANOVA followed by Holm-adjusted planned comparisons. Asterisks indicate comparisons between glucose concentrations within the same treatment (* *p* < 0.05, ** *p* < 0.01); daggers indicate comparisons with the corresponding control (†† *p* < 0.01, ††† *p* < 0.001, †††† *p* < 0.0001); hash symbols indicate comparisons with semaglutide at the corresponding glucose concentration (## *p* < 0.01, ### *p* < 0.001, #### *p* < 0.0001).

The effects of purified M-GLP-1 and M-6HIS-GLP-1 on extracellular insulin accumulation were examined in MIN6 pancreatic β-cells. Cells were treated with either recombinant peptide or semaglutide at 100 nM for 24 h in medium containing 25 or 50 mM glucose. Insulin concentrations were then measured in the conditioned medium (Figure 2).

At 25 mM glucose, insulin concentrations were 101.69 ± 1.26 ng/mL in control cultures, 139.51 ± 1.28 ng/mL with semaglutide, 123.00 ± 1.19 ng/mL with M-GLP-1, and 117.26 ± 1.91 ng/mL with M-6HIS-GLP-1. At 50 mM glucose, the corresponding concentrations were 105.63 ± 0.49, 156.70 ± 0.80, 132.67 ± 1.03, and 127.55 ± 0.60 ng/mL, respectively. Two-way repeated-measures ANOVA showed significant main effects of treatment (F(1.61, 6.42) = 609.21, *p* < 0.001, partial η^2^ = 0.993) and glucose concentration (F(1, 4) = 422.66, *p* < 0.001, partial η^2^ = 0.991), together with a significant treatment × glucose interaction (F(1.57, 6.27) = 6.86, *p* = 0.030, partial η^2^ = 0.632).

At both glucose concentrations, insulin concentrations were significantly higher with semaglutide, M-GLP-1, and M-6HIS-GLP-1 than in the corresponding control cultures. Both recombinant peptides produced lower insulin concentrations than semaglutide. Within each treatment condition, insulin concentrations were higher at 50 mM than at 25 mM glucose, with the largest difference observed for semaglutide. The 24 h measurements reflect insulin accumulated in the conditioned medium rather than an acute glucose-stimulated insulin secretion response.

### 2.3. MIN6 Metabolic Activity Under 25 and 75 mM Glucose Conditions

**Figure 3:**
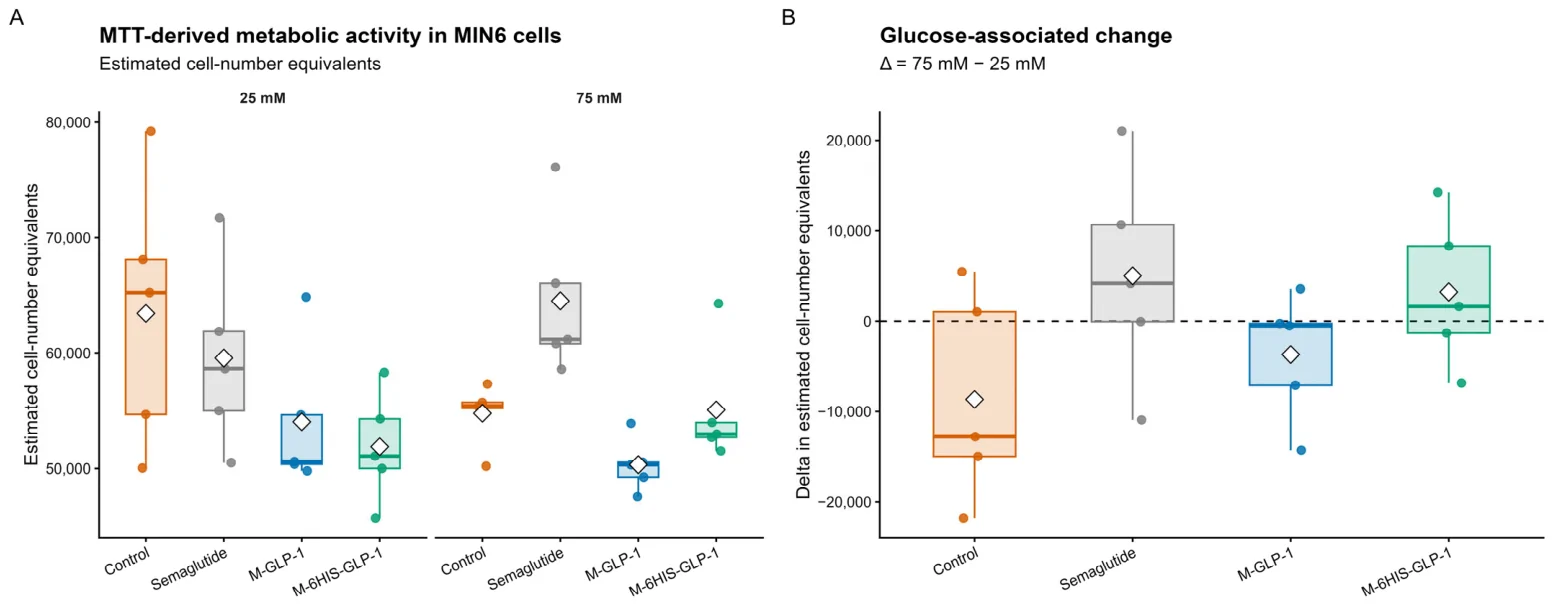
**Metabolic activity measured by MTT in MIN6 cells at 25 and 75 mM glucose.** (**A**) Metabolic activity measured by MTT, expressed as estimated cell number equivalents, after 24 h at 25 or 75 mM glucose in untreated cultures or cultures treated with semaglutide, M-GLP-1, or M-6HIS-GLP-1. (**B**) Change within each experiment, calculated as Δ = value at 75 mM glucose − value at 25 mM glucose. Positive and negative values indicate higher and lower metabolic activity, respectively, at 75 mM glucose. Each point represents the mean of three technical replicate wells from one independent experiment (*n* = 5 independent experiments per condition). Box plots show the median and interquartile range; whiskers indicate the observed range, and white diamonds indicate the mean.

MIN6 cells were incubated for 24 h in medium containing 25 or 75 mM glucose, with or without semaglutide, M-GLP-1, or M-6HIS-GLP-1, to assess metabolic activity under elevated glucose conditions (Figure 3).

MTT-derived metabolic activity, expressed as estimated cell number equivalents, was 63,456 ± 11,501 in control cultures, 59,564 ± 8017 with semaglutide, 54,062 ± 6342 with M-GLP-1, and 51,888 ± 4743 with M-6HIS-GLP-1 at 25 mM glucose. At 75 mM glucose, the corresponding values were 54,808 ± 2704, 64,536 ± 6991, 50,334 ± 2319, and 55,104 ± 5221, respectively.

Two-way repeated-measures ANOVA showed no significant main effect of glucose concentration (F(1, 4) = 0.30, *p* = 0.613), treatment (F(1.45, 5.81) = 4.85, *p* = 0.064), or glucose × treatment interaction (F(2.17, 8.67) = 1.88, *p* = 0.210). Analysis of log-transformed values yielded the same pattern of statistical results. No iso-osmotic control was included. Therefore, the comparison between 25 and 75 mM glucose could not distinguish glucose-specific effects from those associated with increased extracellular osmolarity.

### 2.4. STZ Exposure Reduces MIN6 Metabolic Activity Under All Treatment Conditions

**Figure 4:**
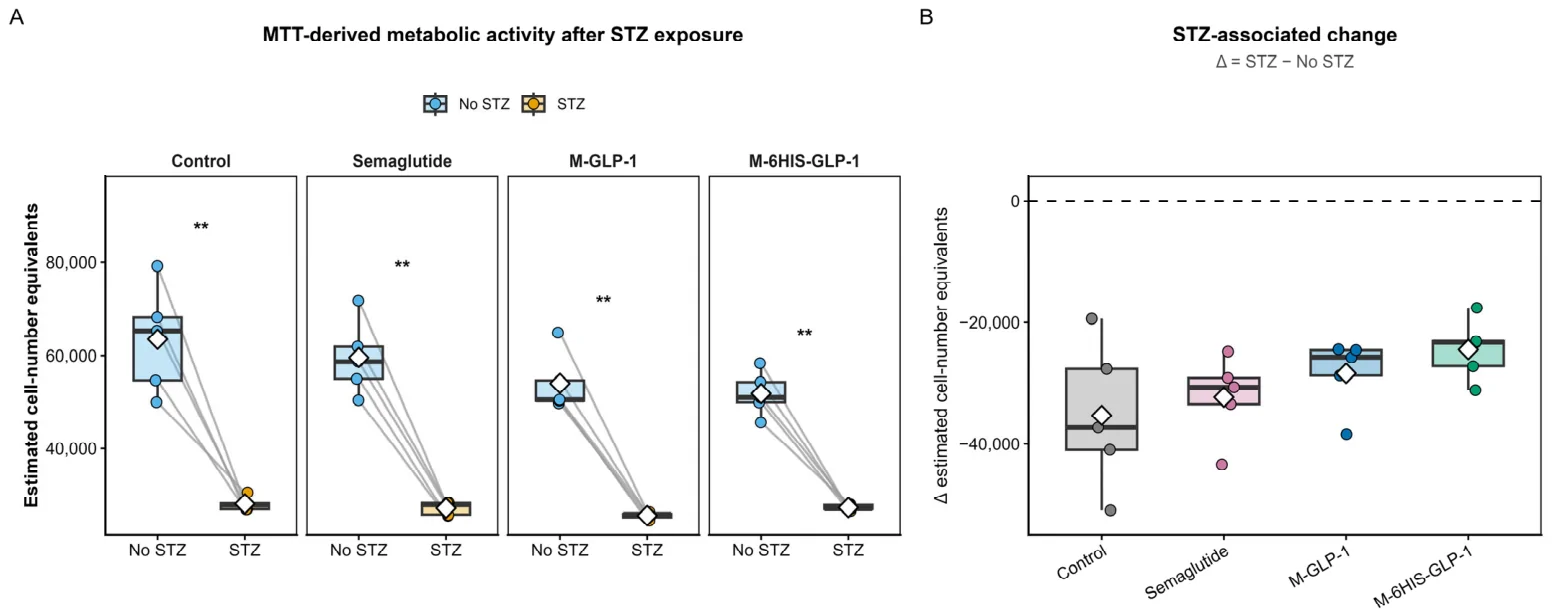
**Effects of STZ exposure on MTT-derived metabolic activity in MIN6 cells.** (**A**) Metabolic activity measured by MTT, expressed as estimated cell number equivalents, after 24 h in the absence or presence of 1 mM STZ and semaglutide, M-GLP-1, or M-6HIS-GLP-1. Each point represents the mean of three technical replicate wells from one independent experiment; lines connect paired observations from the same experiment (*n* = 5 independent experiments per condition). Box plots show the median and interquartile range; whiskers indicate the observed range, and white diamonds indicate the mean. Asterisks indicate Holm-adjusted comparisons between conditions with and without STZ exposure (** *p* < 0.01). (**B**) Change within each experiment calculated as Δ = value after STZ exposure − corresponding value without STZ. Negative values indicate lower metabolic activity following STZ exposure.

To examine the effects of STZ exposure on MIN6 cells, cultures were incubated with 1 mM STZ for 24 h in the absence or presence of semaglutide, M-GLP-1, or M-6HIS-GLP-1 (Figure 4).

STZ exposure reduced MTT-derived metabolic activity under all treatment conditions. Relative to the corresponding cultures without STZ, mean reductions were 55.5% in control cultures, 54.1% with semaglutide, 52.4% with M-GLP-1, and 47.1% with M-6HIS-GLP-1.

Two-way repeated-measures ANOVA showed a significant main effect of STZ exposure (F(1, 4) = 1009.84, *p* < 0.0001, partial η^2^ = 0.996), whereas neither the treatment effect (F(2.18, 8.72) = 2.07, *p* = 0.182, partial η^2^ = 0.341) nor the STZ × treatment interaction (F(1.90, 7.60) = 1.39, *p* = 0.305, partial η^2^ = 0.257) was significant. Holm-adjusted paired comparisons showed lower metabolic activity after STZ exposure within each treatment condition (all adjusted *p* < 0.003).

Analysis of log-transformed values yielded similar results. The effect of STZ remained significant (F(1, 4) = 1908.81, *p* < 0.0001, partial η^2^ = 0.998), whereas the treatment effect (F(2.16, 8.66) = 2.50, *p* = 0.137) and the STZ × treatment interaction (F(1.67, 6.69) = 1.19, *p* = 0.351) were not significant. Although the numerical reduction in metabolic activity was smallest with M-6HIS-GLP-1, no significant treatment-dependent difference in the response to STZ was detected.

### 2.5. Baseline-Adjusted Glucose Concentrations Differ Among STZ-Treated Groups

Following the MIN6 cell experiments with purified M-GLP-1 and M-6HIS-GLP-1, the effects of oral administration of recombinant *L. acidophilus* were evaluated in mice with STZ-induced hyperglycemia.

**Figure 5:**
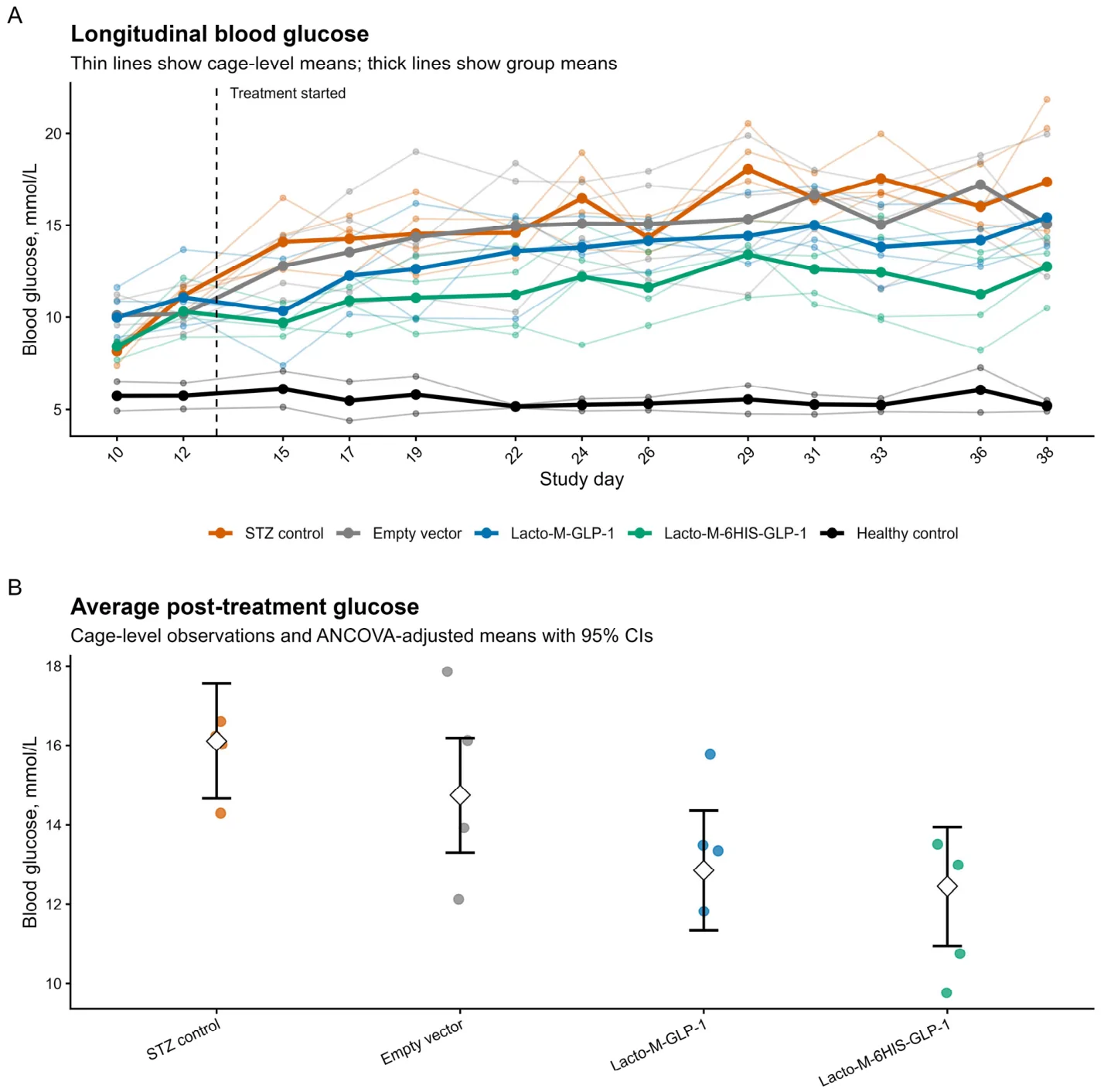
**Longitudinal blood glucose concentrations during treatment.** (**A**) Cage-level mean blood glucose concentrations in STZ control mice, mice receiving *L. acidophilus* carrying the empty vector, Lacto-M-GLP-1, or Lacto-M-6HIS-GLP-1, and untreated healthy controls. Thin lines represent individual cage means, and thick lines represent means across cages within each group. The dashed vertical line indicates treatment initiation on day 13. (**B**) Average post-treatment glucose concentrations across days 15–38 for individual cages in the four STZ-treated groups. Colored points represent cage-level observations; white diamonds and error bars indicate ANCOVA-adjusted group means and 95% confidence intervals after adjustment for mean pretreatment glucose on days 10 and 12. Four cages were analyzed per STZ-treated group.

Blood glucose concentrations were analyzed at the cage level, consistent with cage-level treatment allocation (Figure 5). Across days 15–38, the group × day interaction was not significant (F(30, 120) = 1.07, *p* = 0.387; permutation *p* = 0.380).

Mean post-treatment glucose concentrations, adjusted for pretreatment glucose on days 10 and 12, differed significantly among groups (F(3,11) = 6.85, *p* = 0.0072; permutation *p* = 0.0093). Adjusted mean glucose concentrations were 16.11 mmol/L (95% CI, 14.66–17.56) in the STZ control group, 14.74 mmol/L (95% CI, 13.30–16.18) in the empty-vector group, 12.85 mmol/L (95% CI, 11.33–14.36) in the Lacto-M-GLP-1 group, and 12.44 mmol/L (95% CI, 10.94–13.94) in the Lacto-M-6HIS-GLP-1 group.

Compared with STZ controls, adjusted mean glucose concentrations were lower in the Lacto-M-GLP-1 group (difference, −3.26 mmol/L; simultaneous 95% CI, −6.38 to −0.15; Holm-adjusted *p* = 0.032) and the Lacto-M-6HIS-GLP-1 group (difference, −3.66 mmol/L; simultaneous 95% CI, −6.64 to −0.69; adjusted *p* = 0.014). The empty-vector group did not differ significantly from STZ controls (difference, −1.37 mmol/L; simultaneous 95% CI, −4.37 to 1.64; adjusted *p* = 0.345).

Comparisons with the empty-vector group yielded adjusted differences of −1.89 mmol/L for Lacto-M-GLP-1 (simultaneous 95% CI, −4.89 to 1.10; adjusted *p* = 0.201) and −2.30 mmol/L for Lacto-M-6HIS-GLP-1 (simultaneous 95% CI, −5.38 to 0.79; adjusted *p* = 0.144). The two recombinant-treatment groups did not differ significantly from each other (difference, −0.40 mmol/L; simultaneous 95% CI, −3.64 to 2.84; adjusted *p* = 0.699). The analysis of log-transformed glucose concentrations also showed a significant overall group effect (*p* = 0.0077).

### 2.6. Glucose Profiles During the Intraperitoneal Glucose Tolerance Test

**Figure 6:**
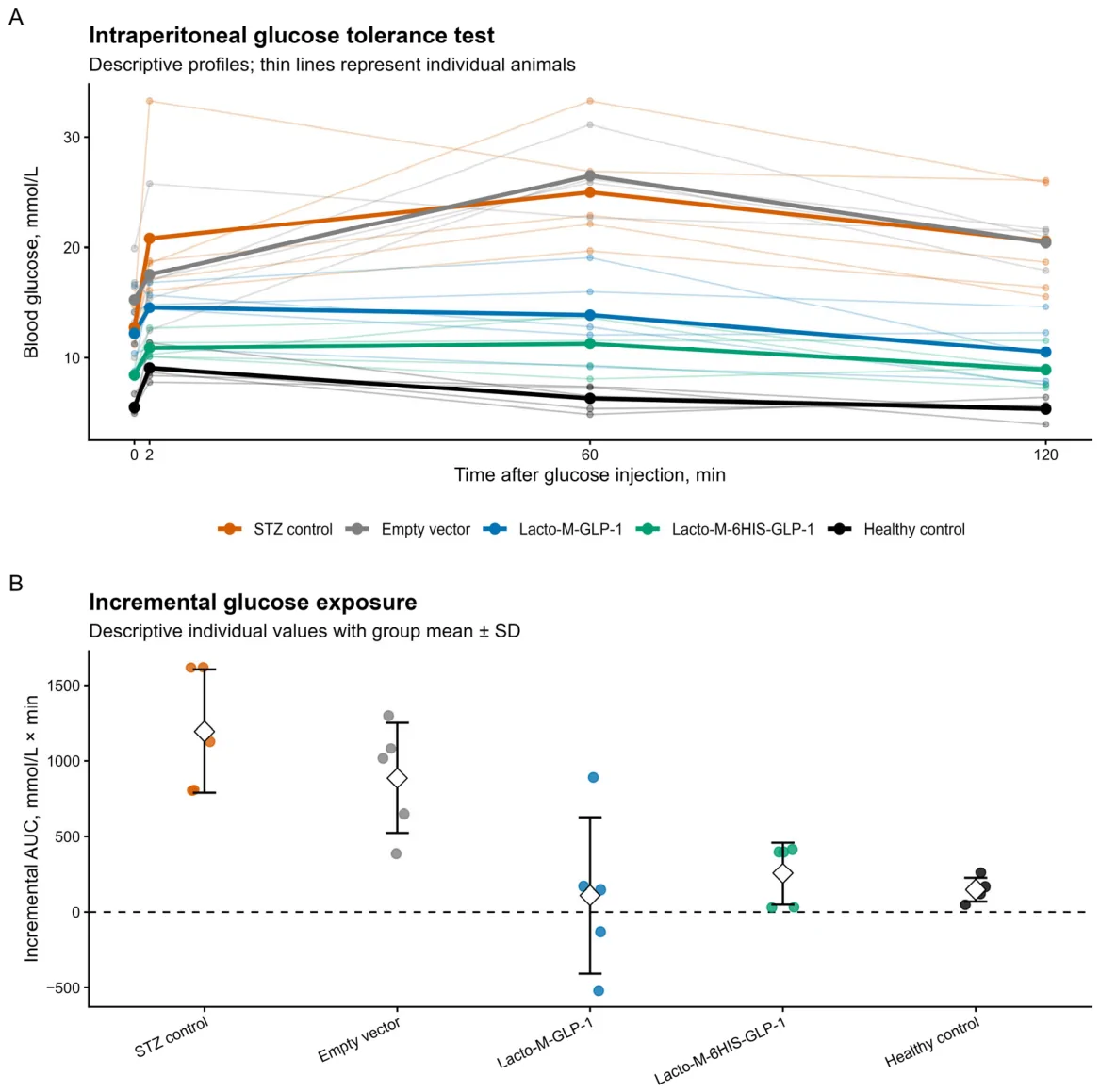
**Glucose responses during the intraperitoneal glucose tolerance test.** (**A**) Blood glucose concentrations before and after intraperitoneal glucose administration on day 38. Thin lines represent individual animals, and thick lines represent group means. Five animals were examined per group; all five animals within each group originated from a single cage. (**B**) Incremental glucose area under the curve during the IPGTT. Each point represents one animal; white diamonds and error bars indicate the group mean ± SD. The dashed horizontal line indicates zero incremental AUC. Because treatment and cage were completely confounded in the IPGTT subset, the data are presented descriptively without treatment-level inferential comparisons.

The IPGTT was performed on day 38 in five mice per group (Figure 6). All five mice within each group originated from a single cage. Therefore, the results were analyzed descriptively.

At 60 min after glucose administration, mean glucose concentrations were 24.98 ± 5.32 mmol/L in the STZ control group, 26.52 ± 3.00 mmol/L in the empty-vector group, 13.84 ± 3.81 mmol/L in the Lacto-M-GLP-1 group, and 11.30 ± 2.57 mmol/L in the Lacto-M-6HIS-GLP-1 group. At 120 min, the corresponding values were 20.52 ± 5.14, 20.42 ± 1.50, 10.54 ± 2.97, and 8.93 ± 1.73 mmol/L, respectively.

Mean incremental area under the curve (iAUC) values were 1196.8 ± 409.3 mmol/L × min in the STZ control group, 887.5 ± 364.0 mmol/L × min in the empty-vector group, 111.8 ± 518.2 mmol/L × min in the Lacto-M-GLP-1 group, and 255.3 ± 203.1 mmol/L × min in the Lacto-M-6HIS-GLP-1 group. Both recombinant-treatment groups showed lower mean glucose concentrations at 60 and 120 min and lower mean iAUC values than the STZ control and empty-vector groups.

### 2.7. Body-Weight Change Differs Among STZ-Treated Groups

**Figure 7:**
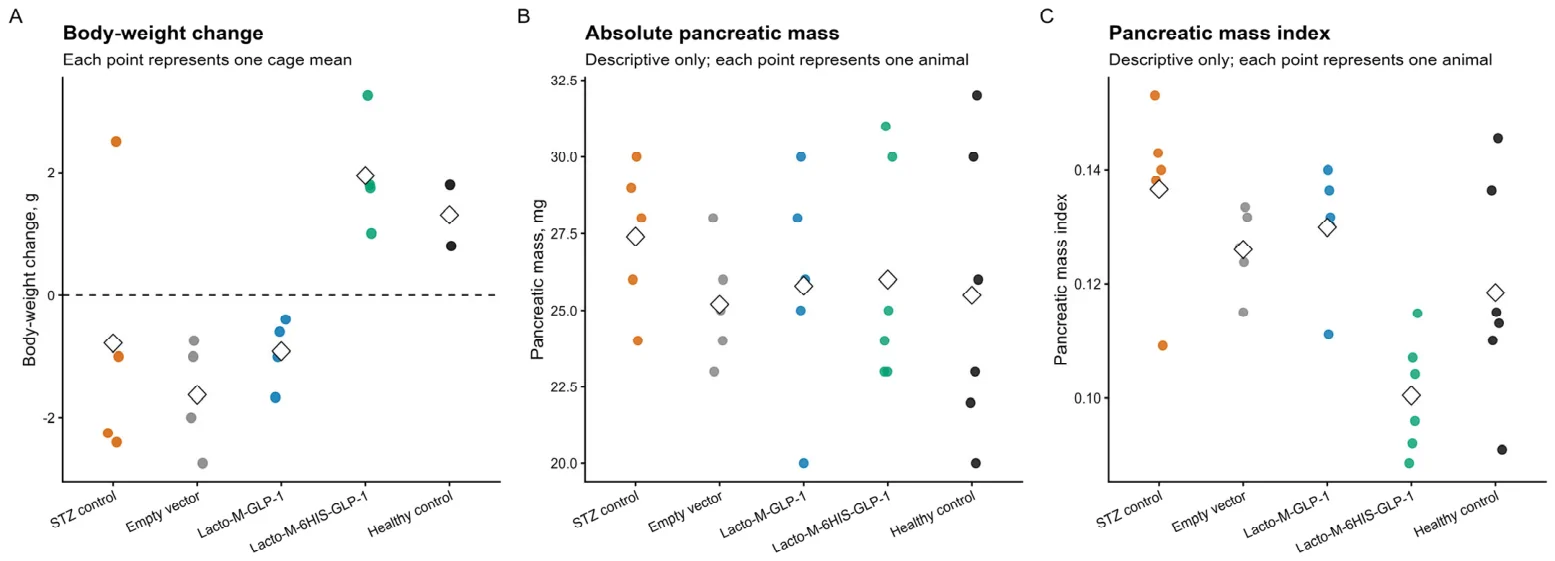
**Body-weight change and pancreatic mass parameters.** (**A**) Mean body-weight change within each cage from pretreatment to day 39. Each colored point represents one cage mean, and white diamonds indicate group means. Four cages were analyzed per STZ-treated group; healthy controls are shown descriptively. Cage-level body-weight change was analyzed using Welch’s one-way ANOVA followed by Games–Howell comparisons. The dashed horizontal line indicates zero body-weight change. (**B**) Absolute pancreatic mass at terminal assessment. (**C**) Pancreatic mass index normalized to terminal body weight. In (**B**,**C**), each point represents one animal, and white diamonds indicate group means. Pancreatic mass endpoints are shown descriptively because the available subsets did not provide independent cage replication across all treatment groups.

Body-weight change from pretreatment to day 39 was analyzed using cage-level means (*n* = 4 cages per STZ-treated group; Figure 7A). Mean changes were −0.79 ± 2.28 g in the STZ control group, −1.63 ± 0.92 g in the empty-vector group, −0.92 ± 0.56 g in the Lacto-M-GLP-1 group, and +1.95 ± 0.94 g in the Lacto-M-6HIS-GLP-1 group. Body-weight change differed among the four groups (Welch’s ANOVA, F(3, 6.25) = 9.71, *p* = 0.009).

Games–Howell comparisons showed greater body-weight change in the Lacto-M-6HIS-GLP-1 group than in the empty-vector group (difference, 3.58 g; 95% CI, 1.29–5.86; *p* = 0.007) and the Lacto-M-GLP-1 group (difference, 2.87 g; 95% CI, 0.83–4.90; *p* = 0.013). The difference from STZ controls was not statistically significant (2.74 g; 95% CI, −2.29 to 7.76; *p* = 0.260), and no other pairwise comparison reached statistical significance. Pretreatment body weight did not differ significantly among groups (*p* = 0.764). ANCOVA of terminal cage-level body weight adjusted for pretreatment weight also identified a group effect (F(3, 11) = 5.37, *p* = 0.016), with a significant difference between Lacto-M-6HIS-GLP-1 and the empty-vector group (adjusted difference, 3.75 g; Bonferroni-adjusted 95% CI, 0.51–6.99; Holm-adjusted *p* = 0.020).

Pancreatic mass was measured in five STZ-control mice from one cage, five empty-vector mice from two cages, five Lacto-M-GLP-1 mice from one cage, and six Lacto-M-6HIS-GLP-1 mice from two cages (Figure 7B,C). These endpoints were summarized descriptively because of insufficient independent cage replication. Mean pancreatic mass was 27.4 ± 2.4 mg in the STZ control group, 25.2 ± 1.9 mg in the empty-vector group, 25.8 ± 3.8 mg in the Lacto-M-GLP-1 group, and 26.0 ± 3.6 mg in the Lacto-M-6HIS-GLP-1 group. The corresponding pancreatic mass indices were 0.137 ± 0.016, 0.126 ± 0.007, 0.130 ± 0.011, and 0.100 ± 0.010, respectively.

### 2.8. Relative Islet Area and Density Are Lower in STZ-Treated Groups than in Healthy Controls

**Figure 8:**
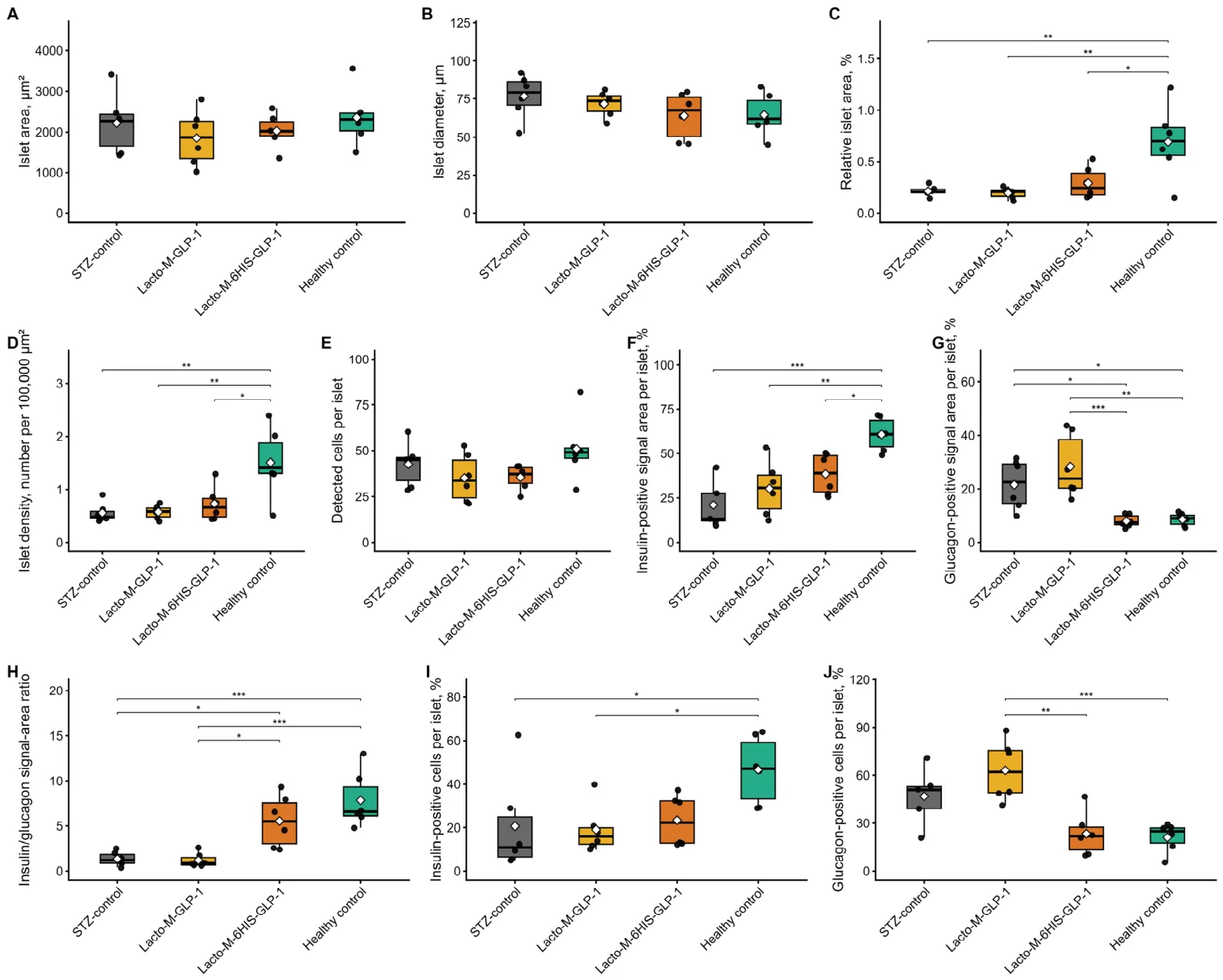
**Pancreatic islet morphometric and endocrine characteristics.** (**A**) Islet area, (**B**) islet diameter, (**C**) relative islet area, (**D**) islet density, (**E**) number of detected cells per islet, (**F**) insulin-positive signal area, (**G**) glucagon-positive signal area, (**H**) ratio of insulin-positive to glucagon-positive signal areas, (**I**) percentage of insulin-positive cells, and (**J**) percentage of glucagon-positive cells. Each point represents a summary value for one animal. Pairwise comparisons are indicated: * *p* < 0.05, ** *p* < 0.01, *** *p* ≤ 0.001. Exact adjusted *p*-values are provided in Supplementary Table S1. Six animals were analyzed per group unless a measurement for an individual endpoint was unavailable (*n* = 5 for the STZ control group for insulin-positive signal area and percentage of glucagon-positive cells). The histological analyses were exploratory.

Pancreatic islet morphometry was assessed in healthy controls and STZ-treated mice (Figure 8A–E and Supplementary Table S1). Six animals per group were analyzed, encompassing 1433 identified islets. The empty-vector group could not be included in the histological analysis because the corresponding pancreatic samples had been lost before analysis.

The mean islet area did not differ significantly among groups (one-way ANOVA, *p* = 0.564), with values of 2218 ± 735 µm^2^ in the STZ control group, 1853 ± 675 µm^2^ in the Lacto-M-GLP-1 group, 2029 ± 418 µm^2^ in the Lacto-M-6HIS-GLP-1 group, and 2354 ± 691 µm^2^ in healthy controls. No significant group differences were detected in the mean islet diameter (*p* = 0.306) or the number of detected cells per islet (*p* = 0.142).

The relative islet area differed among groups (*p* = 0.00082). Mean values were 0.216 ± 0.050% in the STZ control group, 0.197 ± 0.055% in the Lacto-M-GLP-1 group, 0.294 ± 0.150% in the Lacto-M-6HIS-GLP-1 group, and 0.693 ± 0.356% in healthy controls. The relative islet area was lower in the STZ control (*p* = 0.0023), Lacto-M-GLP-1 (*p* = 0.0015), and Lacto-M-6HIS-GLP-1 (*p* = 0.0108) groups than in healthy controls, with no significant differences among the three STZ-treated groups.

Islet density also differed among groups (*p* = 0.00087). Mean values were 0.550 ± 0.184, 0.567 ± 0.129, 0.725 ± 0.325, and 1.50 ± 0.65 islets per 100,000 µm^2^ of pancreatic tissue in the STZ control, Lacto-M-GLP-1, Lacto-M-6HIS-GLP-1, and healthy-control groups, respectively. Islet density was lower in the STZ control (*p* = 0.0018), Lacto-M-GLP-1 (*p* = 0.0021), and Lacto-M-6HIS-GLP-1 (*p* = 0.0108) groups than in healthy controls, whereas no significant differences were detected among the STZ-treated groups.

### 2.9. Lower Glucagon-Positive Signal Area and Higher Insulin-to-Glucagon Signal-Area Ratio in the Lacto-M-6HIS-GLP-1 Group

**Figure 9:**
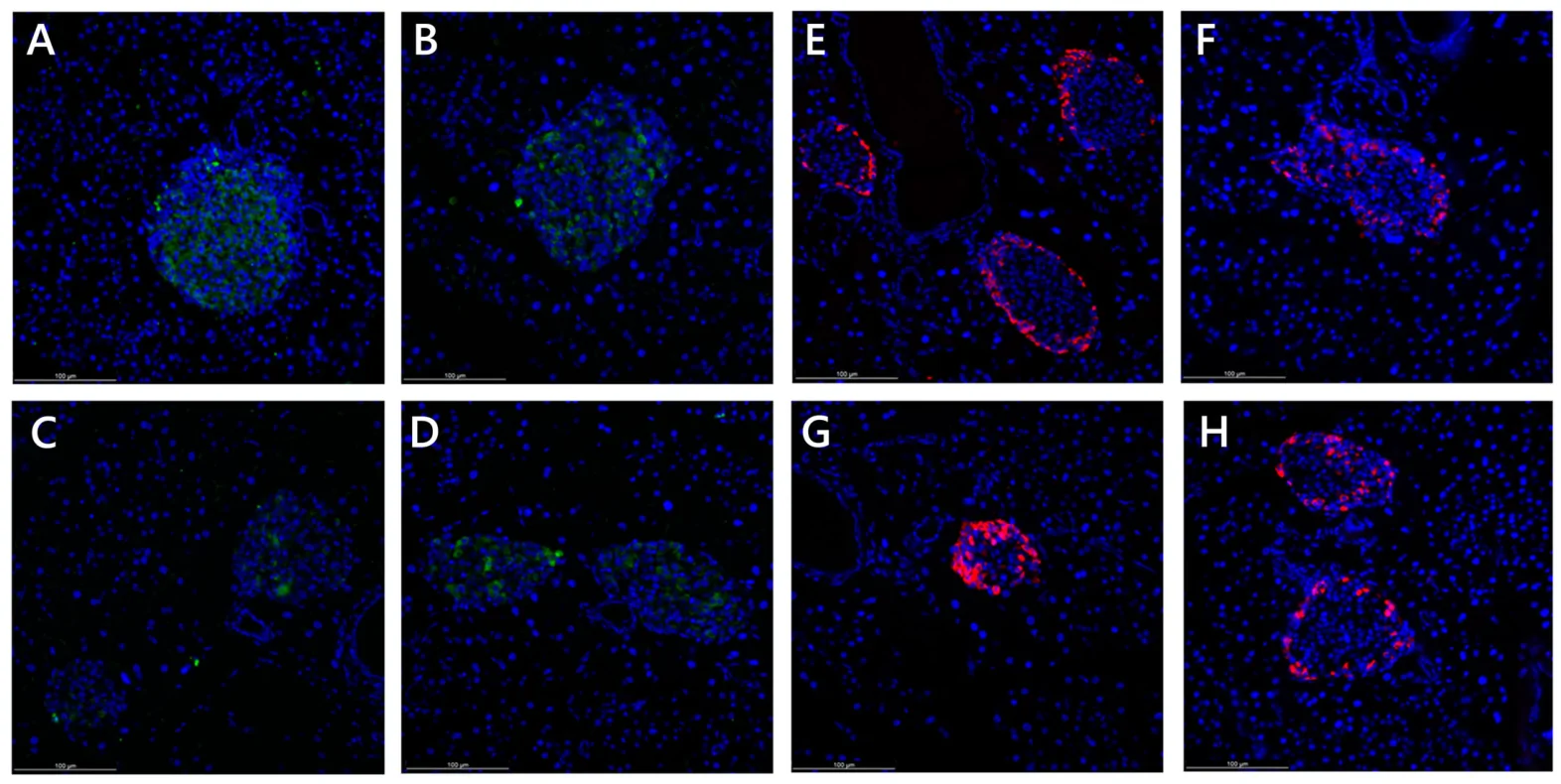
**Representative immunofluorescence staining of insulin and glucagon in pancreatic islets.** Insulin and glucagon were detected in separate adjacent pancreatic sections and are pseudocolored green and red, respectively; nuclei were counterstained with 4′,6-diamidino-2-phenylindole (DAPI; blue). Insulin staining is shown for (**A**) healthy controls, (**B**) STZ controls, (**C**) mice receiving Lacto-M-GLP-1, and (**D**) mice receiving Lacto-M-6HIS-GLP-1. Glucagon staining is shown for (**E**) healthy controls, (**F**) STZ controls, (**G**) mice receiving Lacto-M-GLP-1, and (**H**) mice receiving Lacto-M-6HIS-GLP-1. The scale bar represents 100 µm.

Insulin- and glucagon-positive cell proportions and signal areas were quantified in the histologically analyzed groups (Figure 8F–J and Supplementary Table S1). Representative immunofluorescence images are shown in Figure 9.

The percentage of insulin-positive cells per islet differed among groups (one-way ANOVA, *p* = 0.0221). Mean values were 20.7 ± 22.3% in the STZ control group, 19.1 ± 10.9% in the Lacto-M-GLP-1 group, 23.3 ± 11.7% in the Lacto-M-6HIS-GLP-1 group, and 46.5 ± 15.4% in healthy controls. Compared with healthy controls, the percentage of insulin-positive cells was lower in the STZ control (*p* = 0.0459) and Lacto-M-GLP-1 (*p* = 0.0319) groups. The Lacto-M-6HIS-GLP-1 group did not differ significantly from healthy controls (*p* = 0.0825), the STZ control group (*p* = 0.9909), or the Lacto-M-GLP-1 group (*p* = 0.9653).

The insulin-positive signal area also differed among groups (*p* = 0.00030). Mean values were 20.9 ± 13.9% in the STZ control group, 30.3 ± 15.3% in the Lacto-M-GLP-1 group, 38.5 ± 11.5% in the Lacto-M-6HIS-GLP-1 group, and 60.9 ± 9.5% in healthy controls. The insulin-positive signal area was lower than in healthy controls in the STZ control (*p* = 0.0003), Lacto-M-GLP-1 (*p* = 0.0026), and Lacto-M-6HIS-GLP-1 (*p* = 0.0297) groups. Neither mice receiving Lacto-M-GLP-1 nor mice receiving Lacto-M-6HIS-GLP-1 differed significantly from STZ-treated control mice.

The glucagon-positive signal area differed among groups (*p* = 0.00028). Mean values were 21.5 ± 9.3% in the STZ control group, 28.2 ± 11.9% in the Lacto-M-GLP-1 group, 7.85 ± 2.30% in the Lacto-M-6HIS-GLP-1 group, and 8.45 ± 2.29% in healthy controls. The glucagon-positive signal area was lower in the Lacto-M-6HIS-GLP-1 group than in the STZ control (*p* = 0.0285) and Lacto-M-GLP-1 (*p* = 0.0010) groups. No significant difference was observed between mice receiving Lacto-M-6HIS-GLP-1 and healthy control mice (*p* = 0.9991).

The percentage of glucagon-positive cells per islet also differed among groups (*p* = 0.00035). This percentage was lower in the Lacto-M-6HIS-GLP-1 group than in the Lacto-M-GLP-1 group (*p* = 0.0014), whereas the comparisons with STZ controls (*p* = 0.0874) and healthy controls (*p* = 0.9934) were not statistically significant.

The ratio of insulin-positive to glucagon-positive signal areas differed among groups (*p* < 0.0001). Mean values were 1.35 ± 0.78 in the STZ control group, 1.20 ± 0.80 in the Lacto-M-GLP-1 group, 5.53 ± 2.84 in the Lacto-M-6HIS-GLP-1 group, and 7.85 ± 3.10 in healthy controls. The ratio was higher in the Lacto-M-6HIS-GLP-1 group than in the STZ controls (*p* = 0.0162) and the Lacto-M-GLP-1 group (*p* = 0.0124), with no significant difference from healthy controls (*p* = 0.2829). The corresponding omnibus and selected pairwise sensitivity analyses yielded similar statistical results (Supplementary Table S2).

## 3. Discussion

The present study evaluated GLP-1 variants with *N*-terminal extensions, M-GLP-1 and M-6HIS-GLP-1, as purified peptides in MIN6 cells and as recombinant products expressed in orally administered *L. acidophilus*. Both purified peptides increased insulin concentrations in MIN6 conditioned medium after 24 h. In STZ-treated mice, administration of *L. acidophilus* expressing M-GLP-1 (Lacto-M-GLP-1) or M-6HIS-GLP-1 (Lacto-M-6HIS-GLP-1) was associated with lower average blood glucose concentrations than in STZ controls. However, neither group differed significantly from mice receiving *L. acidophilus* carrying the empty vector. Mice receiving Lacto-M-6HIS-GLP-1 also showed greater body-weight change and differences in pancreatic glucagon staining. However, neither Lacto-M-GLP-1 nor Lacto-M-6HIS-GLP-1 was associated with significant recovery of the measured pancreatic islet morphometric parameters.

Both recombinant peptides developed in the present study differ from native GLP-1(7–37) at the *N*-terminus, a region that is critical for GLP-1R activation. Structural studies have shown that the *N*-terminal residues of GLP-1 interact with the transmembrane receptor core and stabilize its active conformation [11,12,14]. Nevertheless, certain substitutions and chemical modifications in this region can preserve GLP-1R agonism [15,16]. In the present study, AlphaFold 3 predicted broadly similar receptor-bound conformations for native GLP-1 and both modified peptides, despite differences in the calculated interface areas. In turn, purified M-GLP-1 and M-6HIS-GLP-1 increased insulin concentrations in MIN6 conditioned medium after prolonged incubation. These findings suggest that the *N*-terminal extensions did not prevent a cellular response to the peptide preparations. However, insulin concentrations measured in conditioned medium after 24 h reflect accumulation during incubation rather than acute glucose-stimulated insulin secretion [31,32]. Direct GLP-1R signaling assays, such as measurements of cAMP responses, are needed to establish whether M-GLP-1 and M-6HIS-GLP-1 activate GLP-1R [14,15].

GLP-1R activation has been associated with reduced β-cell apoptosis and improved β-cell survival in experimental models [4,33,34]. However, neither M-GLP-1 nor M-6HIS-GLP-1 significantly attenuated the reduction in MIN6 metabolic activity after STZ exposure. Protective effects of GLP-1R agonists have also been demonstrated in cultured pancreatic β-cell lines, including semaglutide-treated INS-1 cells exposed to glucolipotoxic stress and liraglutide-treated MIN6 cells exposed to inflammatory stress [35,36]. In contrast, semaglutide did not significantly attenuate the STZ-associated reduction in MIN6 metabolic activity in the present study. These differences indicate that the protective response observed with GLP-1R agonists depends on the experimental model and conditions. Neither recombinant peptide significantly altered metabolic activity under the 75 mM glucose condition. As MTT reduction reflects cellular metabolic activity rather than apoptosis or cell survival directly [37], these findings do not demonstrate protection against β-cell injury. The high-glucose experiment also lacked an iso-osmotic control, limiting the separation of glucose-dependent effects from those associated with increased osmolarity.

The longitudinal glucose analysis provided the main comparison among the STZ-treated groups. After adjustment for pretreatment glucose concentrations, mice receiving Lacto-M-GLP-1 or Lacto-M-6HIS-GLP-1 had lower average post-treatment glucose concentrations than STZ controls. However, neither group differed significantly from mice receiving *L. acidophilus* carrying the empty vector. Thus, the results did not demonstrate an additional glucose-lowering effect attributable to M-GLP-1 or M-6HIS-GLP-1 expression.

Previous studies have shown that lactic acid bacteria can influence metabolic outcomes independently of recombinant GLP-1 expression. In diabetic rats, *L. paracasei* not expressing GLP-1 reduced blood glucose, whereas expression of pentameric GLP-1 did not produce a significant additional effect [30]. An isogenic *L. paracasei* carrier also affected adiposity independently of GLP-1 secretion [29], while non-engineered *L. acidophilus* improved glucose-related outcomes in HFD/STZ-treated mice [22]. These findings suggest that *L. acidophilus* itself may contribute to the glucose changes observed in mice receiving Lacto-M-GLP-1 or Lacto-M-6HIS-GLP-1.

The IPGTT also showed lower mean glucose concentrations at later time points and lower mean iAUC values in mice receiving Lacto-M-GLP-1 or Lacto-M-6HIS-GLP-1 than in STZ controls and mice receiving empty-vector *L. acidophilus*. Glucose concentrations at individual time points reflect different phases of the glucose response, whereas iAUC summarizes the overall response during the test [38,39]. However, all animals tested within each group originated from a single cage. Therefore, the IPGTT results were descriptive and could not independently establish treatment effects.

GLP-1R-dependent metabolic effects have been demonstrated in other bacterial systems producing GLP-1. Recombinant *L. lactis* producing GLP-1 improved glucose tolerance through a GLP-1R-dependent mechanism and stimulated insulin secretion from wild-type islets but not from GLP-1R-deficient islets [23]. These findings provide evidence that GLP-1 produced by recombinant bacteria can retain receptor-mediated activity.

The *L. acidophilus* constructs expressing M-GLP-1 or M-6HIS-GLP-1 differed from bacterial systems designed to secrete GLP-1-derived peptides. Both peptides were expressed intracellularly, and the constructs lacked a dedicated secretion signal. In contrast, *L. gasseri* secreting a GLP-1 fusion peptide and *L. plantarum* secreting modified GLP-1 improved glucose regulation in db/db mice [27,28]. Recombinant *L. lactis* producing GLP-1 analogues has also been shown to support intestinal peptide uptake and distribution to extraintestinal tissues [40]. Although expression of M-GLP-1 and M-6HIS-GLP-1 was detected in *L. acidophilus* lysates, peptide release, integrity, and availability after oral administration of Lacto-M-GLP-1 or Lacto-M-6HIS-GLP-1 were not assessed.

If released in the intestine, M-GLP-1 and M-6HIS-GLP-1 could influence glucose regulation through local neural or epithelial pathways without substantial systemic absorption. Vagal afferents expressing GLP-1R participate in glucose regulation, and experimental manipulation of these neurons alters glucose tolerance in mice [41]. In another study, administration of commensal bacteria secreting GLP-1(1–37) induced glucose-responsive insulin production in intestinal epithelial cells and improved glucose regulation in diabetic rats [24]. These findings suggest possible intestinal mechanisms of GLP-1 action, although their involvement in mice receiving Lacto-M-GLP-1 or Lacto-M-6HIS-GLP-1 was not investigated.

Pancreatic histology showed that the reductions in relative islet area and islet density associated with STZ treatment were not reversed in mice receiving Lacto-M-GLP-1 or Lacto-M-6HIS-GLP-1. Islet size and cellularity did not differ significantly among the STZ-treated groups included in the histological analysis. Neither insulin-positive cell percentage nor insulin-positive signal area differed significantly between mice receiving Lacto-M-GLP-1 or Lacto-M-6HIS-GLP-1 and STZ controls. Similarly, Duan et al. reported no recovery of pancreatic β-cell counts in diabetic rats receiving bacteria secreting GLP-1(1–37) [24]. In contrast, other studies using recombinant bacteria producing GLP-1-derived peptides have reported changes in pancreatic islet morphology or β-cell-related measures [26,28,40]. The present findings therefore provide no evidence of recovery in the assessed pancreatic islet morphometric parameters following administration of Lacto-M-GLP-1 or Lacto-M-6HIS-GLP-1.

The main differences in pancreatic endocrine staining involved glucagon. Mice receiving Lacto-M-6HIS-GLP-1 had a lower glucagon-positive signal area and a higher insulin-positive-to-glucagon-positive signal-area ratio than STZ controls and mice receiving Lacto-M-GLP-1. Suppression of glucagon secretion is an established component of GLP-1 physiology, and changes in pancreatic glucagon staining have also been reported following administration of bacteria producing modified GLP-1 [3,28]. However, glucagon-positive staining reflects hormone immunoreactivity in pancreatic tissue rather than glucagon secretion, and the insulin-to-glucagon signal-area ratio was calculated from adjacent tissue sections. Furthermore, because mice receiving empty-vector *L. acidophilus* were not included in the histological analysis, these differences cannot be attributed specifically to M-6HIS-GLP-1 expression.

Cage-level analysis showed differences in body-weight change among the STZ-treated groups. Mean body-weight change was positive in the Lacto-M-6HIS-GLP-1 group and negative in the STZ control, empty-vector *L. acidophilus*, and Lacto-M-GLP-1 groups. The difference was significant relative to mice receiving empty-vector *L. acidophilus* or Lacto-M-GLP-1, but not relative to STZ controls. Pharmacological GLP-1R agonists generally reduce body weight in obesity [10], whereas weight loss in STZ-induced hyperglycemia may reflect insulin deficiency and increased catabolism [6]. Administration of *L. acidophilus* has also been associated with greater body weight in HFD/STZ-treated mice [22]. The weight gain observed in mice receiving Lacto-M-6HIS-GLP-1 could therefore reflect reduced catabolism or other changes in energy balance, although food intake and body composition were not assessed. Despite the differences in body-weight change, longitudinal glucose concentrations did not differ significantly between mice receiving Lacto-M-6HIS-GLP-1 and Lacto-M-GLP-1.

The present study has several limitations. Treatment was assigned to existing cages without formal randomization. Longitudinal blood glucose and body-weight outcomes were therefore analyzed using cage-level means. Although this approach accounted for shared housing in the statistical analysis, differences between cages may still have influenced group comparisons. The IPGTT included only one cage per group, while cage identifiers were unavailable for the histological subset, preventing adjustment for cage-related clustering.

No formal prospective sample-size calculation was performed. Longitudinal blood glucose and body-weight change were the principal in vivo outcomes, whereas IPGTT and pancreatic histology were exploratory assessments. The limited number of independent cages reduced the precision of between-group comparisons, and the study was not designed to establish equivalence between treatments.

The IPGTT was also limited by sparse sampling during the early glucose response. The first post-injection measurement was obtained as early as the experimental procedure permitted, but no intermediate measurements were collected before the later phase of the test. The importance of sampling intervals for characterizing glucose tolerance has been emphasized in methodological studies [38,39]. Insulin and glucagon concentrations were not measured during the test, limiting interpretation of the hormonal responses underlying the observed glucose profiles.

The glucose and body-weight responses observed in this STZ-induced model cannot be directly extrapolated to obesity-associated, insulin-resistant T2D. In C57BL/6J mice, repeated low-dose STZ administration has been associated with more pronounced hyperglycemia, impaired glucose tolerance, and pancreatic islet injury in males than in females [42]. As only male mice were studied, whether the responses to Lacto-M-GLP-1 and Lacto-M-6HIS-GLP-1 would be similar in females remains unknown.

Finally, the administered dose of *L. acidophilus* was not systematically optimized. In a murine model of experimental colitis, the effects of *L. acidophilus* varied with the administered dose, and higher doses did not consistently produce greater benefits [43]. Although these findings were obtained in a different disease model, they indicate that bacterial dose can influence treatment outcomes. For Lacto-M-GLP-1 and Lacto-M-6HIS-GLP-1, the administered dose may also affect the amount of intracellularly expressed peptide potentially available for release in the intestine. As only one dose was tested, dose-dependent effects on glucose regulation could not be assessed. Gastrointestinal persistence of the bacteria, plasmid stability, bacterial lysis, peptide release and integrity, and intestinal or systemic exposure were also not investigated. These factors are important for the oral delivery of peptides [17,18].

In the MIN6 experiments, GLP-1R signaling was not directly assessed. The MTT assay measures cellular metabolic activity rather than apoptosis or cell number [37], while the 75 mM glucose experiment lacked an iso-osmotic control. Mannitol controls have been used in MIN6 studies to distinguish glucose-dependent responses from effects of increased extracellular osmolarity [31].

Endotoxin concentrations were not quantified in the recombinant peptide preparations produced in *E. coli*, but both preparations tested negative in a qualitative gel-clot TAL assay. No dedicated step to remove endotoxin was performed during chromatographic purification. However, the purification procedures included anion-exchange chromatography, which has been shown to reduce endotoxin contamination in other protein purification systems [44,45]. Since endotoxin removal was not directly evaluated and sample-specific interference with the TAL assay was not assessed, residual lipopolysaccharide cannot be excluded [46]. Its possible contribution to the MIN6 responses should therefore be considered when interpreting the effects of purified M-GLP-1 and M-6HIS-GLP-1.

## 4. Materials and Methods

### 4.1. Structural Modeling and Analysis of Peptide–GLP-1R Complexes

Structural models of GLP-1R complexes with native human GLP-1(7–37), M-GLP-1, and M-6HIS-GLP-1 were generated using AlphaFold 3 [47]. Five predictions were generated for each peptide–receptor complex.

Structural analyses were performed in UCSF ChimeraX 1.10.1. GLP-1R and the peptide were treated as separate chains. Intermolecular hydrogen bonds were identified using the ChimeraX H-bonds algorithm with relaxed geometric criteria, using a distance tolerance of 0.4 Å and an angular tolerance of 20°. Only hydrogen bonds between the peptide and receptor chains were included.

The peptide–receptor interaction interface was quantified as buried solvent-accessible surface area using the ‘measure buried area’ function in ChimeraX. Confidence metrics from the models, including ipTM and inter-chain predicted alignment error, were obtained from the corresponding AlphaFold 3 outputs.

For each peptide, one prediction was selected for visualization based on proximity to the median interface characteristics, with model confidence metrics also taken into account. GLP-1R structures were aligned using ChimeraX MatchMaker. Interface characteristics were summarized across the five predictions generated for each peptide.

The five predictions for each peptide–receptor complex were used to characterize variation in model-derived interface metrics. Statistical comparisons were restricted to these computational predictions rather than independent experimental measurements of peptide–receptor binding.

### 4.2. Design of the GLP-1 Peptide Variants and Expression Plasmids

Two recombinant GLP-1 variants with *N*-terminal extensions were evaluated. M-GLP-1 consisted of native GLP-1(7–37) preceded by the initiating methionine: MHAEGTFTSDVSSYLEGQAAKEFIAWLVKGRG. M-6HIS-GLP-1 additionally contained a hexahistidine (6×His) extension between the initiating methionine and native GLP-1(7–37): MHHHHHHHAEGTFTSDVSSYLEGQAAKEFIAWLVKGRG.

The expression constructs were designed in this study and subsequently synthesized and assembled by Evrogen JSC (Moscow, Russia) according to the authors’ specifications. For *L. acidophilus*, the constructs were based on the constitutive *Lactobacillus* expression vector pTRK892 [20]. Each expression cassette contained a synthetic ribosome binding site, an ATG initiation codon, the coding sequence for GLP-1(7–37) with or without an *N*-terminal 6×His extension, and tandem translational stop codons (TAA–TGA), inserted between the EcoRI and NotI restriction sites. The coding sequences for the GLP-1 variants were expressed under the control of the Ppgm promoter. The resulting plasmids were designated pTRK892-M-GLP-1 and pTRK892-M-6HIS-GLP-1.

For *Escherichia coli*, the corresponding expression constructs were generated using the pET-21a(+) vector backbone. The coding sequences for M-GLP-1 and M-6HIS-GLP-1 were optimized for codon usage in *E. coli* and cloned between the NdeI and XhoI restriction sites. The codon for translation initiation was provided by the NdeI cloning site. Each insert contained tandem stop codons (TAA–TGA) immediately upstream of the vector-encoded *C*-terminal sequence, preventing translation of the His-tag derived from the vector. The expressed products therefore corresponded to the intended M-GLP-1 or M-6HIS-GLP-1 sequences without an additional *C*-terminal affinity tag. The resulting plasmids were designated pET-21a(+)-M-GLP-1 and pET-21a(+)-M-6HIS-GLP-1.

Neither *L. acidophilus* construct contained a secretion signal peptide or sequence for surface anchoring. The recombinant peptides were therefore designed for intracellular expression rather than active extracellular secretion. Release of intact recombinant peptide from bacterial cells, peptide integrity after release, intestinal exposure, and systemic availability were not measured in the present study.

### 4.3. Cultivation of Bacterial Cultures

*E. coli* BL21(DE3) was used for recombinant peptide production. *L. acidophilus* NCFM served as the host for constitutive expression of the oral bacterial constructs. *E. coli* was cultured in Luria–Bertani (LB) medium at 37 °C with shaking at 200 rpm. *L. acidophilus* was cultured statically in de Man–Rogosa–Sharpe (MRS) broth at 37 °C. Antibiotic selection was performed as required: ampicillin at 100 µg/mL for *E. coli* carrying pET-21a(+)-based constructs and erythromycin at 5 µg/mL for *L. acidophilus*.

### 4.4. Transformation of the Expression Hosts

pTRK892-M-GLP-1 and pTRK892-M-6HIS-GLP-1 were introduced into electrocompetent *L. acidophilus* by electroporation, and transformants were selected on MRS agar containing 5 µg/mL erythromycin [48]. pET-21a(+)-M-GLP-1 and pET-21a(+)-M-6HIS-GLP-1 were transformed into *E. coli* BL21(DE3) cells made chemically competent using CaCl_2_, and transformants were selected on LB agar containing 100 µg/mL ampicillin.

### 4.5. Recombinant Peptide Expression in E. coli and Purification

*E. coli* BL21(DE3) transformants were cultured in LB medium containing 100 µg/mL ampicillin, and recombinant peptide expression was induced with 200 µM isopropyl β-D-1-thiogalactopyranoside (IPTG). Expression was assessed by Tricine–SDS-PAGE according to the Schägger method [49] (Supplementary Figure S1).

M-GLP-1 was purified under denaturing conditions in 8 M urea by sequential anion exchange chromatography on Q-Sepharose and diethylaminoethyl (DEAE)-Sepharose (Cytiva, Uppsala, Sweden). M-6HIS-GLP-1 was purified by Ni-Sepharose affinity chromatography with stepwise imidazole elution (Merck, Darmstadt, Germany), followed by anion-exchange purification on Q-Sepharose [50]. Electrophoretic profiles and apparent molecular masses were examined using the same Tricine–SDS-PAGE method (Supplementary Figure S1). Peptide concentrations were determined by measurement of absorbance at 280 nm using an SF-101 spectrophotometer (Akvilon, Moscow, Russia). GLP-1 immunoreactivity in bacterial lysates was measured using a Human GLP-1 ELISA Kit (E-EL-H6025, Elabscience Biotechnology Inc., Wuhan, China) according to the manufacturer’s instructions. Endotoxin contamination of the purified peptide preparations was assessed using a gel-clot *Tachypleus tridentatus*–lysate (TAL) assay (Bioendo Technology Co., Xiamen, China) according to the manufacturer’s instructions. The stated assay sensitivity was 0.03 EU/mL. Neither preparation formed a gel under the assay conditions, and the assay controls produced the expected results.

Fractions containing the target products were pooled, dialyzed using a membrane with a 3 kDa molecular weight cutoff, lyophilized (ProfLyo T50.6, ProfLab, St. Petersburg, Russia), reconstituted in sterile water, and passed through 0.22-µm filters (Corning Inc., Corning, NY, USA). The purified peptide preparations were stored at 4 °C until use.

### 4.6. Preparation and Quality Control of L. acidophilus Preparations

*L. acidophilus* NCFM carrying pTRK892-M-GLP-1, pTRK892-M-6HIS-GLP-1, or the corresponding empty vector was cultured under the conditions described above. Expression of the recombinant products was evaluated in bacterial lysates by Tricine–SDS-PAGE. Samples were normalized to equivalent colony-forming units (CFUs), and recombinant and empty-vector preparations were analyzed in parallel. Low-molecular-weight bands consistent with the expected recombinant products were detected in the corresponding bacterial preparations (Supplementary Figure S1). GLP-1 immunoreactivity was measured using a Human GLP-1 ELISA Kit (E-EL-H6025, Elabscience Biotechnology Inc., Wuhan, China) according to the manufacturer’s instructions.

Bacterial preparations were lyophilized using a ProfLyo T50.6 freeze dryer (ProfLab, St. Petersburg, Russia). One production and lyophilization batch of each bacterial preparation was used throughout the animal experiment. Viability after lyophilization was determined by rehydration of representative aliquots, serial dilution, and plating on selective MRS agar. For administration, suspensions were adjusted to 1 × 10^7^ CFU/mL, and 100 µL was administered per mouse, corresponding to 1 × 10^6^ CFU per administration.

### 4.7. MIN6 Cell Culture

MIN6 mouse pancreatic β-cells were used for all cellular experiments. Cells were maintained in high-glucose Dulbecco’s Modified Eagle Medium (DMEM; 4.5 g/L glucose, approximately 25 mM) supplemented with 15% fetal bovine serum, 2 mM L-glutamine, 50 µM β-mercaptoethanol, 100 U/mL penicillin, and 100 µg/mL streptomycin. Cells were maintained at 37 °C in a humidified atmosphere containing 5% CO_2_ and passaged at 80–90% confluence using 0.25% trypsin–EDTA. Cell numbers were determined using an automated cell counter TC20 (Bio-Rad, Hercules, CA, USA) before plating. Cells between passages 5 and 20 were used for experiments.

### 4.8. Measurement of Insulin Concentrations in MIN6 Conditioned Medium

MIN6 cells were seeded in 96-well plates at 1 × 10^4^ cells per well and allowed to attach for 24 h. Treatment medium had the same composition as the routine culture medium described above and contained either 25 mM glucose or glucose supplemented to a final concentration of 50 mM. Cells were treated with vehicle (control medium without semaglutide or recombinant peptide), semaglutide, M-GLP-1, or M-6HIS-GLP-1 at a final concentration of 100 nM for 24 h.

Conditioned media were collected and cleared by centrifugation. Insulin concentrations were measured using a mouse insulin ELISA kit (E-EL-M2614, Elabscience Biotechnology Inc., Wuhan, China) according to the manufacturer’s instructions. Three technical replicates were averaged to obtain one value per condition within each independent experiment. Five independent experiments were performed, with all treatment × glucose combinations included in each experiment.

### 4.9. MTT Assay

MIN6 cellular metabolic activity was assessed using the 3-(4,5-Dimethylthiazol-2-yl)-2,5-diphenyltetrazolium bromide (MTT) assay. Cells were seeded in 96-well plates at 5 × 10^4^ cells per well and allowed to attach for 24 h.

Following experimental treatment, MTT stock solution (5 mg/mL) was added at 10% of the assay volume, and cells were incubated for 4 h at 37 °C. Formazan crystals were dissolved in dimethyl sulfoxide (DMSO). Absorbance was measured at 570 nm using a Synergy H1 microplate reader (BioTek Instruments, Winooski, VT, USA), with background correction at 630 nm. Absorbance values were converted to estimated cell-number equivalents using a calibration curve generated from serially diluted MIN6 cells under the same assay conditions.

### 4.10. MIN6 Response to High-Glucose Conditions

MIN6 cells were incubated for 24 h in medium containing either 25 or 75 mM glucose and were treated with vehicle, semaglutide, M-GLP-1, or M-6HIS-GLP-1 at 100 nM. The treatment medium otherwise had the same composition as the routine culture medium.

Metabolic activity was quantified using the MTT assay. Five independent experiments were performed, with all glucose × treatment combinations included in each experiment. Three technical replicate wells were averaged to obtain one experiment-level value. No iso-osmotic control was included.

### 4.11. MIN6 Response to STZ-Induced Cellular Stress

MIN6 cells were exposed to STZ (Sigma-Aldrich, St. Louis, MO, USA), freshly dissolved in sterile water, at a final concentration of 1 mM for 24 h [51] and were treated with vehicle, semaglutide, M-GLP-1, or M-6HIS-GLP-1 at 100 nM. Corresponding conditions without STZ were included for each treatment. The treatment medium otherwise had the same composition as the routine culture medium.

Metabolic activity was quantified using the MTT assay. Five independent experiments were performed, with all STZ exposure × treatment combinations included in each experiment. Three technical replicate wells were averaged to obtain one experiment-level value.

### 4.12. Animals, Housing, and Experimental Design

Male C57BL/6J mice (*n* = 90, 8 weeks old; initial body weight approximately 20 g) were obtained from the Stolbovaya branch of the Scientific Center of Biomedical Technologies of the Federal Medical and Biological Agency of Russia. Animals were allowed a 14-day acclimatization period before the start of experimental procedures.

Mice were housed at 20–24 °C and 45–65% relative humidity under a 12 h light/12 h dark cycle (lights on 08:00–20:00). A standard laboratory rodent diet (“CHARA”, Favorit LLC, Sergiev Posad, Russia) and drinking water purified by reverse osmosis were provided ad libitum except during fasting periods specified in the protocol. Environmental enrichment was provided. Cages were cleaned and bedding replaced once weekly. Animals were monitored daily for general health and welfare. Humane endpoints included marked body-weight loss, severe lethargy, dehydration, and inability to independently access food or water.

Ten mice formed the untreated healthy control group, and 80 received STZ. Eight STZ-treated mice did not meet the glycemic eligibility criterion and were excluded before treatment allocation. The remaining 72 mice were assigned to four experimental groups of 18 animals each: STZ control, *L. acidophilus* carrying the empty vector, Lacto-M-GLP-1, and Lacto-M-6HIS-GLP-1.

Treatment was assigned at the cage level without formal randomization. Existing cages were allocated to the four STZ-treated groups based on cage size to obtain 18 animals per group, without regrouping cage-mates. Allocation was not based on individual glucose concentrations, body weight, or other measured characteristics. Each group comprised four cages, with 5, 4, 5, and 4 mice in the STZ control group; 5, 5, 4, and 4 in the empty-vector group; 5, 3, 5, and 5 in the Lacto-M-GLP-1 group; and 4, 4, 5, and 5 in the Lacto-M-6HIS-GLP-1 group.

The study lasted 39 days. The intraperitoneal glucose tolerance test (IPGTT) was performed on day 38, followed by euthanasia and tissue collection on day 39. All procedures were approved by the Institutional Ethics Committee of the Koltzov Institute of Developmental Biology of the Russian Academy of Sciences (Protocol No. 74, 14 September 2023).

### 4.13. Induction of STZ-Associated Hyperglycemia and Eligibility Criteria

STZ, freshly dissolved in sterile 0.9% sodium chloride solution for injection, was administered intraperitoneally at 50 mg/kg once daily on days 1–5.

Blood glucose was measured on days 10 and 12. The prespecified glycemic eligibility range used in the experiment was >8 and <15 mmol/L. Mice were eligible for treatment allocation when at least one of the two measurements fell within this range; mice for which neither measurement met the criterion were excluded before treatment allocation.

### 4.14. Administration of Bacterial Preparations and Longitudinal Monitoring

Administration of bacterial preparations or control solution began on day 13. Preparations were administered by oral gavage three times weekly (Monday, Wednesday, and Friday). The Lacto-M-GLP-1, Lacto-M-6HIS-GLP-1, and empty-vector groups received 1 × 10^6^ CFU in 100 µL per administration. Oral administration of *L. acidophilus* has previously been investigated across a broad dose range in murine models, including 10^4^–10^8^ CFU per 10 g of body weight [43]. The dose of 1 × 10^6^ CFU per administration was selected as an intermediate dose within the reported range. The STZ control group received 100 µL sterile 0.9% NaCl.

Blood glucose was measured using a CONTOUR TS glucometer (Ascensia Diabetes Care, Basel, Switzerland) on days 7, 10, 12, 15, 17, 19, 22, 24, 26, 29, 31, 33, 36, and 38. The fasting glucose measurement before injection obtained during the IPGTT on day 38 was recorded separately from the routine longitudinal measurement.

Body weight was recorded one day before the first administration and on day 39 before euthanasia.

### 4.15. Intraperitoneal Glucose Tolerance Test

The IPGTT was performed on day 38 in five mice per experimental group, all originating from a single cage within each group. Animals were fasted for 6–8 h before testing. Glucose was administered intraperitoneally at 1 g/kg body weight using a 200 mg/mL solution.

Blood glucose was measured immediately before glucose administration and at 2, 60, and 120 min thereafter using a CONTOUR TS glucometer (Ascensia Diabetes Care, Basel, Switzerland). The 2 min time point was the earliest post-injection measurement feasible within the experimental workflow, whereas the 60 and 120 min measurements characterized later glucose responses.

### 4.16. Change in Body Weight and Pancreatic Mass

Body weight was measured using laboratory scales (OHAUS Corporation, Parsippany, NJ, USA). Individual change in body weight was calculated as terminal body weight minus pretreatment body weight. For treatment-level analysis, individual body-weight values were averaged within each cage.

Pancreatic mass was determined in a subset of animals from each experimental group. After excision, the pancreas was gently blotted to remove excess liquid and weighed before fixation. The pancreatic mass index was calculated as follows:

(1) $$\mathrm{Pancreatic}\;\mathrm{mass}\;\mathrm{index},\;\%=\frac{\mathrm{Pancreatic}\;\mathrm{mass}\;(g)}{\mathrm{Terminal}\;\mathrm{body}\;\mathrm{weight}\;(g)}\times 100$$

Absolute pancreatic mass was reported as the unnormalized organ-weight measure, whereas the pancreatic mass index was reported as a body-weight-normalized measure. Because the pancreatic-mass subsets did not provide independent cage replication across all treatment groups, pancreatic mass and pancreatic mass index were summarized descriptively and were not subjected to treatment-level inferential comparisons.

### 4.17. Tissue Collection and Histological Processing

On day 39, animals were euthanized by an intraperitoneal overdose of tribromoethanol (Sigma-Aldrich, St. Louis, MO, USA; 550 mg/kg), followed by cervical dislocation as a secondary physical method to ensure death.

For histological analysis, six mice were randomly selected from each of four groups: healthy control, STZ control, Lacto-M-GLP-1, and Lacto-M-6HIS-GLP-1. Selection was performed before tissue processing without reference to individual glycemic measurements. Pancreatic samples were also collected from the empty-vector group but were lost due to technical issues before histological analysis.

Pancreata were rinsed in physiological saline and fixed for 24 h at 4 °C in freshly prepared 4% paraformaldehyde (Sigma-Aldrich, St. Louis, MO, USA) in phosphate-buffered saline (PBS). Samples were dehydrated through ascending ethanol concentrations, cleared in xylene, and embedded in paraffin embedding medium (BioVitrum, St. Petersburg, Russia). Serial sections (5 µm thick) were prepared throughout the pancreatic lobes using a Microm HM430 microtome (Thermo Fisher Scientific, Waltham, MA, USA).

### 4.18. Immunofluorescence Staining

Pancreatic sections were deparaffinized and rehydrated through a descending ethanol series. Tissue autofluorescence was reduced by incubation for 20 min in Sudan Black B solution (Merck, Darmstadt, Germany).

For each animal, two pancreatic levels were selected from the serial section series based on maximal pancreatic tissue area. The two selected section pairs represented pancreatic levels separated by approximately 80–100 µm. Within each pair, adjacent serial sections were used separately for insulin and glucagon immunostaining because both primary antibodies were raised in rabbit.

Glucagon was detected using a rabbit polyclonal anti-glucagon antibody (Sigma, SAB4501137; 1:500), and insulin using a rabbit polyclonal anti-insulin antibody (ELK Biotechnology, Wuhan, China; ES5905; 1:200). Primary antibodies were diluted in PBS (PanEco, Moscow, Russia) containing 10% bovine serum albumin (PAA Laboratories GmbH, Pasching, Austria) and 1% Triton X-100 (AppliChem GmbH, Darmstadt, Germany). Sections were incubated with primary antibodies for 18 h at 12 °C.

Sections were incubated with a goat anti-rabbit IgG (H+L) Cross-Adsorbed Secondary Antibody conjugated to Alexa Fluor 594 (A-11012; Invitrogen, Carlsbad, CA, USA; 1:1000) for 2 h at room temperature. Nuclei were counterstained with DAPI (Biotium Inc., Fremont, CA, USA; 1:5000) for 10 min. Sections were mounted using Mowiol mounting medium (Calbiochem, Merck, Darmstadt, Germany).

Sections processed without primary antibody were used to determine background fluorescence and support threshold selection for subsequent image analysis.

### 4.19. Microscopy and Image Acquisition

Images were acquired using a Leica THUNDER DMi8 microscope (Leica Microsystems GmbH, Wetzlar, Germany) equipped with a high-resolution digital camera and Leica LAS X software version 3.10.0. Exposure time, detector gain, and other acquisition settings were kept constant within each fluorescence channel.

For each animal, panoramic images were acquired from two selected pancreatic section pairs at 5× magnification. All identifiable pancreatic islets within the analyzed sections were subsequently imaged at 20× magnification using transmitted light, DAPI fluorescence (435 nm), and Alexa Fluor 594 hormone-specific immunofluorescence (594 nm).

Images acquired using transmitted light were captured together with fluorescence images and used as an additional anatomical reference for pancreatic islet contour delineation. Insulin and glucagon signals were recorded on separate adjacent serial sections within each selected pair. The sections within each insulin–glucagon pair were selected to be as close to each other as possible in the serial section sequence.

Fluorescence images were acquired in grayscale. Pseudocolors were assigned during image visualization and figure preparation to facilitate interpretation: DAPI was shown in blue, insulin immunofluorescence was pseudocolored green, and glucagon immunofluorescence was pseudocolored red.

### 4.20. Quantitative Pancreatic Morphometry

Quantitative image analysis was performed in QuPath version 0.5.0 using the StarDist extension. Two pancreatic levels were analyzed per animal.

Pancreatic tissue area was manually delineated on 5× panoramic images, excluding non-pancreatic tissue, adipose tissue, lymph nodes, and other irrelevant regions. Pancreatic islets were manually delineated on 20× images using transmitted light, DAPI, and hormone-specific fluorescence as anatomical references. Nuclei were detected using StarDist, and approximate cell boundaries were generated using the QuPath workflow for cell detection.

Insulin- and glucagon-positive cells were classified using QuPath object classifiers based on predefined 594 nm fluorescence intensity thresholds. Hormone-positive signal areas were quantified using corresponding pixel classifiers. Thresholds were established using sections processed without primary antibody and background regions and were applied consistently within each staining experiment. Custom Groovy scripts were used for batch processing and data extraction.

The recorded endpoints included islet area, islet diameter, total detected cell number, numbers of insulin- and glucagon-positive cells, and insulin- and glucagon-positive signal areas.

Islet density was calculated as

(2) $$\mathrm{Islet}\;\mathrm{density}=\frac{\mathrm{Number}\;\mathrm{of}\;\mathrm{detected}\;\mathrm{islets}}{\mathrm{Analyzed}\;\mathrm{pancreatic}\;\mathrm{tissue}\;\mathrm{area},\;{\mathrm{µm}}^{2}}\times 100,000$$

and expressed as the number of islets per 100,000 µm^2^ of analyzed pancreatic tissue.

The relative islet area was calculated as

(3) $$\mathrm{Relative}\;\mathrm{islet}\;\mathrm{area},\;\%=\frac{\mathrm{Total}\;\mathrm{islet}\;\mathrm{area}}{\mathrm{Analyzed}\;\mathrm{pancreatic}\;\mathrm{tissue}\;\mathrm{area},\;{\mathrm{µm}}^{2}}\times 100$$

Insulin- and glucagon-positive signal areas were expressed as percentages of the corresponding islet area:

(4) $$\mathrm{Hormone}-\mathrm{positive}\;\mathrm{signal}\;\mathrm{area},\;\%\;=\frac{\mathrm{Hormone}-\mathrm{positive}\;\mathrm{signal}\;\mathrm{area}}{\mathrm{Total}\;\mathrm{islet}\;\mathrm{area}}\times 100$$

The percentages of insulin- and glucagon-positive cells were calculated as:

(5) $$\mathrm{Hormone}-\mathrm{positive}\;\mathrm{cells},\;\%\;=\frac{\mathrm{Number}\;\mathrm{of}\;\mathrm{hormone}-\mathrm{positive}\;\mathrm{cells}}{\mathrm{Total}\;\mathrm{number}\;\mathrm{of}\;\mathrm{detected}\;\mathrm{cells}}\times 100$$

For morphometric endpoints derived from tissue sections, values from the two pancreatic levels were averaged within each animal before statistical analysis. The ratio of insulin-positive to glucagon-positive signal areas was calculated by dividing the percentage of insulin-positive signal area calculated for each animal by the corresponding percentage of glucagon-positive signal area. Image files were coded before analysis, and the investigator remained blinded to treatment allocation until measurements for each animal had been finalized.

### 4.21. Statistical Analysis

Statistical analyses were performed in R version 4.6.1 using RStudio [52]. Data processing, statistical analyses, and visualization were performed using the lme4 (version 1.1-37), lmerTest (version 3.2.0), pbkrtest (version 0.5.4), emmeans (version 2.0.0), afex (version 1.5-1), rstatix (version 0.7.3), tidyverse (version 2.0.0), and ggplot2 (version 4.0.3) packages. Unless otherwise specified, data are presented as mean ± standard deviation (SD), and model-based estimates are reported with 95% confidence intervals (CIs). No formal prospective sample-size calculation was performed. Longitudinal blood glucose and body-weight change were the main in vivo outcomes, whereas IPGTT and pancreatic histology were analyzed as exploratory endpoints.

For AlphaFold 3-derived structural metrics, buried peptide–receptor interface area and the number of predicted intermolecular hydrogen bonds were compared among peptide variants using the Kruskal–Wallis test followed by pairwise Wilcoxon rank-sum tests with Holm adjustment for multiple comparisons.

Insulin concentrations in conditioned medium were analyzed using two-way repeated-measures ANOVA, with treatment and glucose concentration as within-experiment factors. The independent experiment was the repeated-measures unit. MTT data were analyzed similarly, using glucose concentration × treatment or STZ exposure × treatment as within-experiment factors. Greenhouse–Geisser correction was applied where appropriate, and effect sizes were expressed as partial η^2^. Planned contrasts of estimated marginal means in the insulin assay were adjusted using the Holm method. For the STZ experiment, paired comparisons between STZ-exposed and unexposed conditions within each treatment were performed using Holm-adjusted paired *t*-tests. Log-transformed MTT data were also analyzed as a sensitivity analysis.

Because treatment was allocated at the cage level, cages were used as independent experimental units for treatment-level analyses of blood glucose and body weight. For longitudinal glucose analysis, individual measurements were averaged within each cage at each observation day. The group × day interaction across days 15–38 was assessed using repeated-measures ANOVA, with group as a between-cage factor and day as a within-cage factor. A supplementary permutation analysis was performed using 10,000 permutations of group labels among complete cage-level glucose profiles.

For the main glucose analysis, mean post-treatment glucose concentration was calculated for each cage by averaging cage-level measurements across days 15–38. Group differences were assessed by ANCOVA, with treatment group as a fixed factor and mean pretreatment glucose on days 10 and 12 as a continuous covariate. Adjusted group means were estimated at the overall mean pretreatment glucose concentration. All six pairwise comparisons among the four STZ-treated groups were evaluated using Holm-adjusted *p*-values and Bonferroni-adjusted simultaneous 95% CIs. The overall group effect was additionally examined using 10,000 permutations of cage-level group labels. A sensitivity analysis was performed by repeating the ANCOVA after log transformation of individual glucose measurements and subsequent cage-level aggregation.

IPGTT glucose profiles and incremental area under the curve (iAUC) were summarized descriptively because all five animals within each experimental group originated from a single cage. iAUC was calculated relative to individual pre-injection glucose concentrations using the trapezoidal rule. Negative incremental areas were retained when glucose concentrations fell below baseline.

Body-weight change was calculated for each animal and averaged within cages. Pretreatment body weight was compared among the four STZ-treated groups using Welch’s one-way ANOVA. Cage-level body-weight changes were analyzed using Welch’s one-way ANOVA followed by Games–Howell pairwise comparisons. As a sensitivity analysis, terminal cage-level body weight was analyzed by ANCOVA with pretreatment cage-level body weight as a continuous covariate. Pairwise comparisons were evaluated using Holm-adjusted *p*-values and Bonferroni-adjusted 95% CIs.

Pancreatic mass and pancreatic mass index were summarized descriptively because the available subsets did not provide sufficient independent cage replication for between-treatment comparisons.

For pancreatic morphometric and endocrine histological endpoints, measurements from the analyzed tissue sections were aggregated at the animal level. Group differences were assessed using one-way ANOVA followed by Tukey-adjusted pairwise comparisons. Benjamini–Hochberg false discovery rate correction was applied to omnibus *p*-values across the ten histological endpoints (Supplementary Table S1). Sensitivity analyses included Welch ANOVA, transformations of selected variables, and permutation tests, with Holm adjustment for pairwise permutation comparisons (Supplementary Table S2). Cage identifiers were unavailable for the histological subset, preventing adjustment for cage-level clustering.

Multiplicity adjustments were applied within the specified comparison families. No study-wide multiplicity correction was applied across distinct outcome families.

## 5. Conclusions

The present study demonstrated that purified M-GLP-1 and M-6HIS-GLP-1 increased insulin concentrations in MIN6 conditioned medium, supporting further investigation of these *N*-terminally extended GLP-1 variants. In STZ-treated mice, administration of *L. acidophilus* expressing either peptide was associated with lower average blood glucose concentrations than in STZ controls. Lacto-M-6HIS-GLP-1 was also associated with greater body-weight change than Lacto-M-GLP-1 and empty-vector *L. acidophilus*, together with differences in pancreatic glucagon-related staining compared with STZ controls and Lacto-M-GLP-1.

These findings demonstrate cellular responses to purified M-GLP-1 and M-6HIS-GLP-1 and metabolic differences in mice receiving *L. acidophilus* expressing these peptides. However, the glucose-lowering effects did not differ significantly from those observed with empty-vector *L. acidophilus*. The contribution of recombinant peptide expression to the observed effects therefore requires further investigation. In particular, studies of peptide release and stability after oral administration, intestinal availability, and GLP-1R-dependent activity are needed to establish the potential of these constructs as a bacterial GLP-1 delivery system.

## Data Availability

Individual-level data supporting the in vivo analyses, including available cage identifiers, together with the R code used for the statistical analyses and figure generation, are publicly available in Zenodo at https://doi.org/10.5281/zenodo.23536079. Additional data are available from the corresponding author upon reasonable request.

## Supplementary Materials

The following supporting information can be downloaded at https://www.mdpi.com/article/10.3390/ijms272110003/s1.

## References

[1] Abel E.D., Gloyn A.L., Evans-Molina C., Joseph J.J., Misra S., Pajvani U.B., Simcox J., Susztak K., Drucker D.J. (2024). Diabetes Mellitus—Progress and Opportunities in the Evolving Epidemic. Cell.

[2] Accili D., Deng Z., Liu Q. (2025). Insulin Resistance in Type 2 Diabetes Mellitus. Nat. Rev. Endocrinol..

[3] Gasbjerg L.S., Nielsen C.K., Suppli M.P., Grøndahl M.F.G., Holst J.J., Knop F.K., Jeppesen P.B., Lund A.B. (2026). Proglucagon-Derived Peptides: Human Physiology and Therapeutic Potential. Physiol. Rev..

[4] Vogt É.L., Kowaltowski A.J. (2026). GLP-1, Pancreatic β-Cells, and Insulin Secretion: What We Know and Where We Need to Go. Diabetes.

[5] Lenzen S. (2008). The Mechanisms of Alloxan- and Streptozotocin-Induced Diabetes. Diabetologia.

[6] Furman B.L. (2021). Streptozotocin-Induced Diabetic Models in Mice and Rats. Curr. Protoc..

[7] Nauck M.A., Meier J.J. (2018). Incretin Hormones: Their Role in Health and Disease. Diabetes Obes. Metab..

[8] Lee A.A., Khotina V.A., Kashirskikh D.A., Voronko O.E., Gasanov V.A., Vasiliev A.V. (2025). Incretin-Based Therapies Through the Decades: Molecular Innovations and Clinical Impact. Med. Sci..

[9] Kaplan J.M., Zaman A., Abushamat L.A. (2024). Curbing the Obesity Epidemic: Should GLP-1 Receptor Agonists Be the Standard of Care for Obesity?. Curr. Cardiol. Rep..

[10] Drucker D.J. (2025). GLP-1-Based Therapies for Diabetes, Obesity and Beyond. Nat. Rev. Drug Discov..

[11] Zhou Q., Zhao F., Zhang Y., Yang D., Wang M.-W. (2025). Structural Pharmacology and Mechanisms of GLP-1R Signaling. Trends Pharmacol. Sci..

[12] Zhang Y., Sun B., Feng D., Hu H., Chu M., Qu Q., Tarrasch J.T., Li S., Sun Kobilka T., Kobilka B.K. (2017). Cryo-EM Structure of the Activated GLP-1 Receptor in Complex with a G Protein. Nature.

[13] Yang D., de Graaf C., Yang L., Song G., Dai A., Cai X., Feng Y., Reedtz-Runge S., Hanson M.A., Yang H. (2016). Structural Determinants of Binding the Seven-Transmembrane Domain of the Glucagon-like Peptide-1 Receptor (GLP-1R). J. Biol. Chem..

[14] Cary B.P., Deganutti G., Zhao P., Truong T.T., Piper S.J., Liu X., Belousoff M.J., Danev R., Sexton P.M., Wootten D. (2022). Structural and Functional Diversity among Agonist-Bound States of the GLP-1 Receptor. Nat. Chem. Biol..

[15] Longwell C.K., Hanna S., Hartrampf N., Sperberg R.A.P., Huang P.-S., Pentelute B.L., Cochran J.R. (2021). Identification of N-Terminally Diversified GLP-1R Agonists Using Saturation Mutagenesis and Chemical Design. ACS Chem. Biol..

[16] Sicinski K.M., Montanari V., Raman V.S., Doyle J.R., Harwood B.N., Song Y.C., Fagan M.P., Rios M., Haines D.R., Kopin A.S. (2021). A Non-Perturbative Molecular Grafting Strategy for Stable and Potent Therapeutic Peptide Ligands. ACS Cent. Sci..

[17] Yu L., Han X., Liu J., Yan X., Li T. (2026). Engineered Bacteria as Living Materials for Oral Delivery of Peptide Drugs. ACS Appl. Mater. Interfaces.

[18] Gelli H.P., Vazquez-Uribe R., Buckley S.T., Andersen J.T., Alexander Sommer M.O. (2025). Advanced Microbiome Therapeutics for Oral Delivery of Peptides and Proteins: Advances, Challenges, and Opportunities. Adv. Drug Deliv. Rev..

[19] Mejía-Caballero A., Marco M.L. (2026). Lactobacilli Biology, Applications and Host Interactions. Nat. Rev. Microbiol..

[20] Duong T., Miller M.J., Barrangou R., Azcarate-Peril M.A., Klaenhammer T.R. (2011). Construction of Vectors for Inducible and Constitutive Gene Expression in Lactobacillus. Microb. Biotechnol..

[21] Rodrigues R.R., Gurung M., Li Z., García-Jaramillo M., Greer R., Gaulke C., Bauchinger F., You H., Pederson J.W., Vasquez-Perez S. (2021). Transkingdom Interactions between Lactobacilli and Hepatic Mitochondria Attenuate Western Diet-Induced Diabetes. Nat. Commun..

[22] Yan F., Li N., Yue Y., Wang C., Zhao L., Evivie S.E., Li B., Huo G. (2019). Screening for Potential Novel Probiotics With Dipeptidyl Peptidase IV-Inhibiting Activity for Type 2 Diabetes Attenuation in Vitro and in Vivo. Front. Microbiol..

[23] Arora T., Wegmann U., Bobhate A., Lee Y.S., Greiner T.U., Drucker D.J., Narbad A., Bäckhed F. (2016). Microbially Produced Glucagon-like Peptide 1 Improves Glucose Tolerance in Mice. Mol. Metab..

[24] Duan F.F., Liu J.H., March J.C. (2015). Engineered Commensal Bacteria Reprogram Intestinal Cells into Glucose-Responsive Insulin-Secreting Cells for the Treatment of Diabetes. Diabetes.

[25] Luo J., Zhang H., Lu J., Ma C., Chen T. (2021). Antidiabetic Effect of an Engineered Bacterium Lactobacillus Plantarum-pMG36e -GLP-1 in Monkey Model. Synth. Syst. Biotechnol..

[26] Hu H., Luo J., Liu Y., Li H., Jin R., Li S., Wei J., Wei H., Chen T. (2023). Improvement Effect of a Next-Generation Probiotic L. Plantarum-pMG36e-GLP-1 on Type 2 Diabetes Mellitus via the Gut-Pancreas-Liver Axis. Food Funct..

[27] Ke Z., Ma Q., Ye X., Jin Y., Wang Y., Zhao X., Su Z. (2025). Oral Delivery of GLP-1 Peptide Using Recombinant Lactobacillus Gasseri for the Treatment of Type 2 Diabetes Mellitus. Microbiol. Spectr..

[28] Wang Q., Guo H., Mao W., Qian X., Liu Y. (2023). The Oral Delivery System of Modified GLP-1 by Probiotics for T2DM. Pharmaceutics.

[29] Ryan P.M., Patterson E., Kent R.M., Stack H., O’Connor P.M., Murphy K., Peterson V.L., Mandal R., Wishart D.S., Dinan T.G. (2017). Recombinant Incretin-Secreting Microbe Improves Metabolic Dysfunction in High-Fat Diet Fed Rodents. Sci. Rep..

[30] Lin Y., Krogh-Andersen K., Pelletier J., Marcotte H., Östenson C.-G., Hammarström L. (2016). Oral Delivery of Pentameric Glucagon-Like Peptide-1 by Recombinant Lactobacillus in Diabetic Rats. PLoS ONE.

[31] Cheruiyot A., Hollister-Lock J., Sullivan B., Pan H., Dreyfuss J.M., Bonner-Weir S., Schaffer J.E. (2023). Sustained Hyperglycemia Specifically Targets Translation of mRNAs for Insulin Secretion. J. Clin. Investig..

[32] Yang L., Chen W. (2023). Insulin Secretion Assays in an Engineered MIN6 Cell Line. MethodsX.

[33] Li Y., Hansotia T., Yusta B., Ris F., Halban P.A., Drucker D.J. (2003). Glucagon-like Peptide-1 Receptor Signaling Modulates Beta Cell Apoptosis. J. Biol. Chem..

[34] Rea N., Ramdass P.V.A.K. (2025). GLP-1 Receptor Agonists and Pancreatic Beta Cell Apoptosis in Diabetes Mellitus: A Systematic Review and Meta-Analysis of Preclinical Studies. Front. Clin. Diabetes Healthc..

[35] Gojani E.G., Wang B., Li D., Kovalchuk O., Kovalchuk I. (2025). Single and Combined Impact of Semaglutide, Tirzepatide, and Metformin on β-Cell Maintenance and Function Under High-Glucose-High-Lipid Conditions: A Comparative Study. Int. J. Mol. Sci..

[36] Ding F., Luo X., Tu Y., Duan X., Liu J., Jia L., Zheng P. (2021). Alpk1 Sensitizes Pancreatic Beta Cells to Cytokine-Induced Apoptosis via Upregulating TNF-α Signaling Pathway. Front. Immunol..

[37] Ghasemi M., Turnbull T., Sebastian S., Kempson I. (2021). The MTT Assay: Utility, Limitations, Pitfalls, and Interpretation in Bulk and Single-Cell Analysis. Int. J. Mol. Sci..

[38] Benedé-Ubieto R., Estévez-Vázquez O., Ramadori P., Cubero F.J., Nevzorova Y.A. (2020). Guidelines and Considerations for Metabolic Tolerance Tests in Mice. Diabetes Metab. Syndr. Obes. Targets Ther..

[39] Moro C., Magnan C. (2025). Revisited Guidelines for Metabolic Tolerance Tests in Mice. Lab Anim..

[40] Huang Y., Lin X., Deng M., Tang Y., Li S., Xu B., Zeng W., Chen Z., Hou X., Lin Z. (2026). Oral Delivery of GLP-1 Analogues by Recombinant Lactococcus Lactis Restores Pancreatic Islet Structure through Intestinal Mucosal Absorption in Diabetic Mice. EBioMedicine.

[41] Borgmann D., Ciglieri E., Biglari N., Brandt C., Cremer A.L., Backes H., Tittgemeyer M., Wunderlich F.T., Brüning J.C., Fenselau H. (2021). Gut-Brain Communication by Distinct Sensory Neurons Differently Controls Feeding and Glucose Metabolism. Cell Metab..

[42] Kim B., Park E.-S., Lee J.-S., Suh J.G. (2025). Sex-Specific Differences in Glucose Metabolism and Pancreatic Function in Streptozotocin-Induced Diabetic Mice: The Protective Role of Estrogen. Biochem. Biophys. Res. Commun..

[43] Chen L.-L., Zou Y.-Y., Lu F.-G., Li F.-J., Lian G.-H. (2013). Efficacy Profiles for Different Concentrations of Lactobacillus Acidophilus in Experimental Colitis. World J. Gastroenterol..

[44] Wespel M., Geiss M., Nägele M., Combé S., Reich J., Studts J., Stolzenberger J. (2022). The Impact of Endotoxin Masking on the Removal of Endotoxin during Manufacturing of a Biopharmaceutical Drug Product. J. Chromatogr. A.

[45] Heimann S., Vail A., Muczenski S., Faude A. (2025). Comparative Analysis of Anion Exchange Membrane Adsorbers for Endotoxin Removal During Ultrafiltration and Diafiltration Buffer Preparation. Eng. Life Sci..

[46] Bonhomme D., Cavaillon J.-M., Werts C. (2024). The Dangerous Liaisons in Innate Immunity Involving Recombinant Proteins and Endotoxins: Examples from the Literature and the *Leptospira* Field. J. Biol. Chem..

[47] Abramson J., Adler J., Dunger J., Evans R., Green T., Pritzel A., Ronneberger O., Willmore L., Ballard A.J., Bambrick J. (2024). Accurate Structure Prediction of Biomolecular Interactions with AlphaFold 3. Nature.

[48] Kong L., Huang Y., Zeng X., Ye C., Wu Z., Guo Y., Pan D. (2022). Effects of Galactosyltransferase on EPS Biosynthesis and Freeze-Drying Resistance of Lactobacillus Acidophilus NCFM. Food Chem..

[49] Schägger H., von Jagow G. (1987). Tricine-Sodium Dodecyl Sulfate-Polyacrylamide Gel Electrophoresis for the Separation of Proteins in the Range from 1 to 100 kDa. Anal. Biochem..

[50] Loughran S.T., Bree R.T., Walls D. (2023). Poly-Histidine-Tagged Protein Purification Using Immobilized Metal Affinity Chromatography (IMAC). Methods Mol. Biol..

[51] Jiang Y., He P., Sheng K., Peng Y., Wu H., Qian S., Ji W., Guo X., Shan X. (2025). The Protective Roles of Eugenol on Type 1 Diabetes Mellitus through NRF2-Mediated Oxidative Stress Pathway. eLife.

[52] R Core Team R: A Language and Environment for Statistical Computing. https://www.R-project.org/.

