# Supplementary material for: Oral Administration of Lactobacillus acidophilus Expressing GLP-1 Peptide Variants with N-Terminal Extensions in Mice with Streptozotocin-Induced Hyperglycemia
Source: Int J Mol Sci. 2026 Nov 8;27(21):10003. doi: 10.3390/ijms272110003 (PMC13764476; doi:10.3390/ijms272110003)
Supplement: Supplementary file 1 [file ijms-27-10003-s001.zip › ijms-4640175-supplementary.pdf]

Supplementary Materials

Oral Administration of *Lactobacillus acidophilus* Expressing GLP-1 Peptide Variants with *N*-Terminal Extensions in Mice with Streptozotocin-Induced Hyperglycemia

Arthur A. Lee, Maria B. Chernysheva, Dmitry A. Kashirskikh, Evgenii S. Ruchko, Olesya O. Klychkova, Victoria A. Khotina*, Tatiana V. Noskova, Vagif A. Gasanov, Ekaterina A. Vorotelyak, Andrey V. Vasiliev

Koltzov Institute of Developmental Biology of Russian Academy of Sciences, 119334 Moscow, Russia; aal1999arth@gmail.com (A.A.L.); m.b.chern@gmail.com (M.B.C.); dim.kashirsckih@gmail.com (D.A.K.); ruchko7@gmail.com (E.S.R.); olesya_k8@mail.ru (O.O.K.); t.noskova@idbras.ru (T.V.N.); vorotelyak@yandex.ru (E.A.V.); gasanovvagif@gmail.com (V.A.G.); a.v.vasilev@idbras.ru (A.V.V.)

* Correspondence: v.a.khotina.bio@gmail.com (V.A.K.)

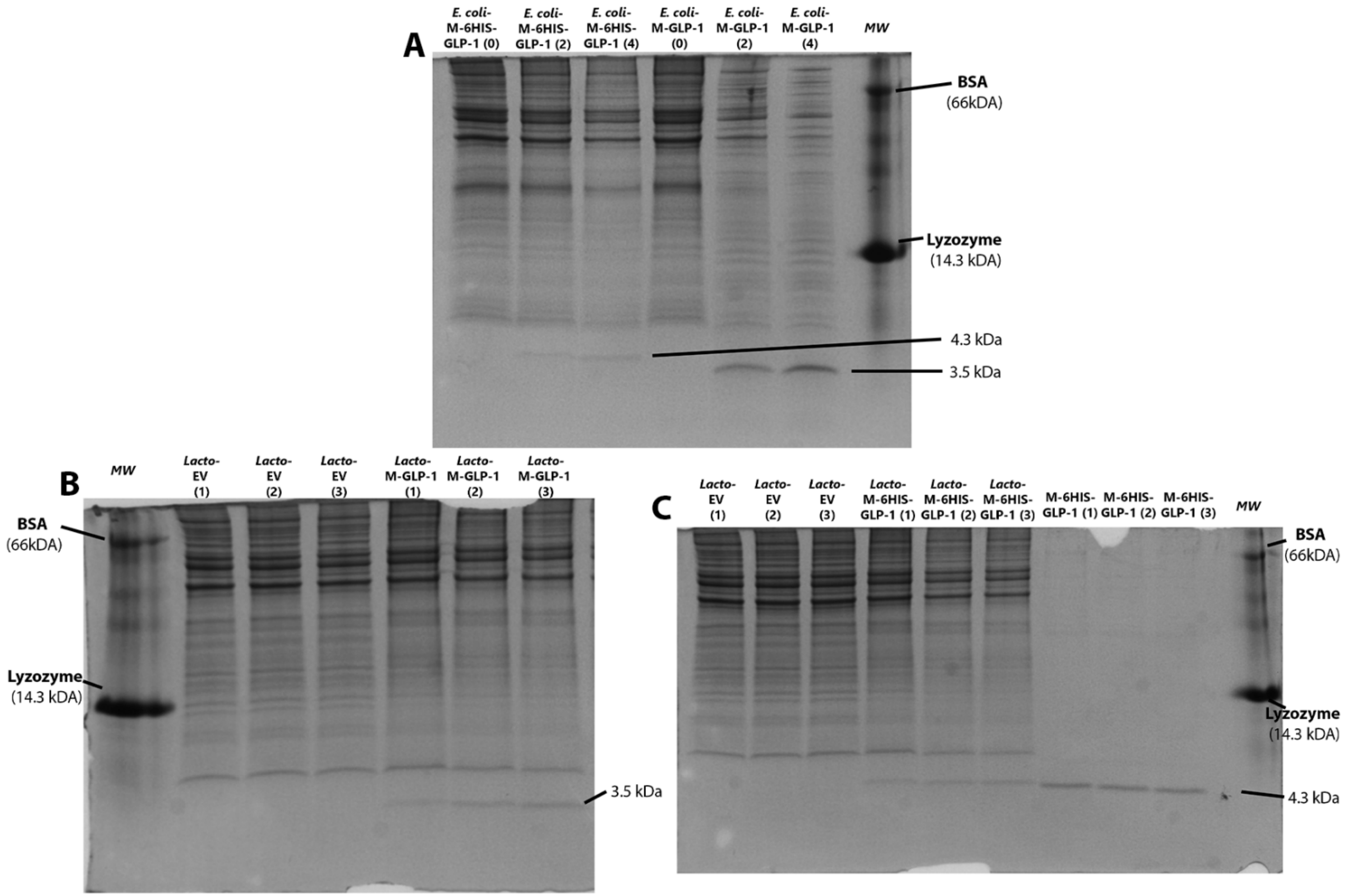

Figure S1. Tricine–SDS-PAGE analysis of recombinant GLP-1 variants during bacterial expression and purification. (A) *E. coli* BL21(DE3) expressing M-6HIS-GLP-1 or M-GLP-1 before induction (0 h) and at 2 and 4 h after induction. **(B)** Analysis of *L. acidophilus* carrying the empty vector (Lacto-EV) or expressing M-GLP-1 (Lacto-M-GLP-1) after 1, 2, and 3 h of incubation in MRS medium. **(C)** Analysis of Lacto-EV or expressing M-6HIS-GLP-1 (Lacto-M-6HIS-GLP-1) after 1, 2, and 3 h of incubation in MRS medium. The three rightmost lanes in panel C represent technical replicates of the M-6HIS-GLP-1 fraction obtained after Ni-Sepharose affinity chromatography. The indicated positions correspond to the expected molecular masses of M-GLP-1 (~3.5 kDa) and M-6HIS-GLP-1 (~4.3 kDa). Bovine serum albumin (BSA, 66 kDa) and lysozyme (14.3 kDa) were used as molecular-mass reference proteins (MW).

Supplementary Table S1. Group-level summary statistics and inferential comparisons of pancreatic islet morphometric and endocrine histological endpoints

Endpoint	STZ-control	Lacto-M-GLP-1	Lacto-M-6HIS-GLP-1	Healthy control	ANOVA *p*	BH-FDR *q*	STZ vs M-GLP-1	STZ vs M-6HIS-GLP-1	STZ vs Healthy	M-GLP-1 vs M-6HIS-GLP-1	M-GLP-1 vs Healthy	M-6HIS vs Healthy
Islet area, μm^2	2218.1 ± 734.8 (n=6)	1852.8 ± 675.3 (n=6)	2028.8 ± 417.8 (n=6)	2354.1 ± 690.9 (n=6)	0.563746	0.563745	0.7591	0.9555	0.9826	0.9638	0.5420	0.8162
Islet diameter, µm	76.7 ± 14.4 (n=6)	71.6 ± 8.2 (n=6)	63.9 ± 15.1 (n=6)	64.5 ± 13.7 (n=6)	0.305857	0.339841	0.9074	0.3603	0.4000	0.7458	0.7879	0.9998
Relative islet area, %	0.216 ± 0.050 (n=6)	0.197 ± 0.055 (n=6)	0.294 ± 0.150 (n=6)	0.693 ± 0.356 (n=6)	0.000822	0.001446	0.9982	0.8998	0.0023	0.8258	0.0015	0.0108
Islet density, per 100,000 μm^2	0.550 ± 0.184 (n=6)	0.567 ± 0.129 (n=6)	0.725 ± 0.325 (n=6)	1.500 ± 0.654 (n=6)	0.000868	0.001446	0.9998	0.8568	0.0018	0.8890	0.0021	0.0108
Detected cells per islet	42.6 ± 11.9 (n=6)	35.2 ± 13.1 (n=6)	35.6 ± 6.5 (n=6)	51.0 ± 17.3 (n=6)	0.142389	0.177986	0.7487	0.7797	0.6700	0.9999	0.1735	0.1911
Insulin-positive signal area, %	20.9 ± 13.9 (n=5)	30.3 ± 15.3 (n=6)	38.5 ± 11.5 (n=6)	60.9 ± 9.5 (n=6)	0.000302	0.000869	0.6123	0.1342	0.0003	0.6871	0.0026	0.0297
Glucagon-positive signal area, %	21.5 ± 9.3 (n=6)	28.2 ± 11.9 (n=6)	7.85 ± 2.30 (n=6)	8.45 ± 2.29 (n=6)	0.000280	0.000869	0.4505	0.0285	0.0379	0.0010	0.0013	0.9991
Insulin-to-glucagon signal-area ratio	1.35 ± 0.78 (n=6)	1.20 ± 0.80 (n=6)	5.53 ± 2.84 (n=6)	7.85 ± 3.10 (n=6)	0.000047	0.000474	0.9994	0.0162	0.0002	0.0124	0.0002	0.2829
Insulin-positive cells, %	20.7 ± 22.3 (n=6)	19.1 ± 10.9 (n=6)	23.3 ± 11.7 (n=6)	46.5 ± 15.4 (n=6)	0.022121	0.031602	0.9981	0.9909	0.0459	0.9653	0.0319	0.0825
Glucagon-positive cells, %	47.0 ± 18.5 (n=5)	63.0 ± 18.8 (n=6)	23.5 ± 13.6 (n=6)	21.2 ± 9.2 (n=6)	0.000348	0.000869	0.3409	0.0874	0.0535	0.0014	0.0008	0.9934

Data are presented as mean ± SD. For section-derived morphometric endpoints, values from the two analyzed pancreatic sections were averaged within each animal before statistical analysis; the animal was used as the experimental unit. One-way ANOVA was used for overall group comparisons, followed by Tukey's multiple-comparisons test. Benjamini–Hochberg false discovery rate (BH-FDR) q-values were calculated across the 10 endpoint-level omnibus tests as a supplementary multiplicity analysis. Bold values indicate $p < 0.05$ or $q < 0.05$. Sample size was six animals per group unless an endpoint-specific measurement was unavailable; n = 5 for the STZ-control group for insulin-positive signal area and glucagon-positive cells. In pairwise comparison labels, M-GLP-1 and M-6HIS denote the Lacto-M-GLP-1 and Lacto-M-6HIS-GLP-1 treatment groups, respectively. These analyses were exploratory and did not account for cage-level clustering because cage identifiers were unavailable for the histological subset.

Supplementary Table S2. Robustness and sensitivity analyses of pancreatic morphometric and endocrine histological endpoints

Table S2A. Robustness of endpoint-level group effects

Endpoint	Primary one-way ANOVA, *p*	Welch ANOVA, *p*	Permutation omnibus test, *p*	Transformation used	Transformed ANOVA, *p*
Islet area	0.5637	0.6460	0.5664	—	—
Islet diameter	0.3059	0.4219	0.3046	—	—
Relative islet area	0.00082	0.0437	0.00119	ln(*x*)	0.00251
Islet density	0.00087	0.0444	0.00128	ln(*x*)	0.00160
Detected cells per islet	0.1424	0.2499	0.1347	—	—
Insulin-positive signal area	0.00030	0.00133	0.00037	logit(proportion)	0.00058
Glucagon-positive signal area	0.00028	0.00422	0.00055	logit(proportion)	<0.0001
Insulin-to-glucagon signal-area ratio	<0.0001	0.00143	0.00016	ln(*x*)	<0.0001
Insulin-positive cells	0.0221	0.0371	0.0234	logit(proportion)	0.0233
Glucagon-positive cells	0.00035	0.00411	0.00070	logit(proportion)	0.00082

Table S2B. Sensitivity analyses of the principal glucagon-related pairwise comparisons

Endpoint	Comparison	Primary Tukey-adjusted *p*	Exact permutation *p*	Holm-adjusted permutation *p*	Tukey *p* after transformation
Glucagon-positive signal area	M-6HIS vs STZ-control	0.0285	0.00649	0.0260	0.0031
	M-6HIS vs M-GLP-1	0.0010	0.00216	0.0130	0.0001
	M-6HIS vs Healthy control	0.9991	0.6580	0.6580	0.9901
Insulin-to-glucagon signal-area ratio	M-6HIS vs STZ-control	0.0162	0.00433	0.0173	0.0012
	M-6HIS vs M-GLP-1	0.0124	0.00649	0.0195	0.0006
	M-6HIS vs Healthy control	0.2829	0.2121	0.4242	0.5818
Glucagon-positive cells	M-6HIS vs STZ-control	0.0874	0.0433	0.1299	0.1253
	M-6HIS vs M-GLP-1	0.0014	0.00433	0.0216	0.0033
	M-6HIS vs Healthy control	0.9934	0.7662	0.7662	0.9902

Sensitivity analyses of pancreatic morphometric and endocrine hormone-related endpoints. The individual animal was used as the experimental unit. Welch ANOVA was used to assess robustness to heterogeneity of variances. Permutation omnibus tests were used as distribution-robust sensitivity analyses. Relative islet area, islet density, and the insulin-to-glucagon signal-area ratio were natural-log transformed where indicated. Percentage variables were converted to proportions and logit-transformed as $\ln[p/(1-p)]$. Pairwise permutation p-values were adjusted using the Holm procedure. Primary inferential results remained those obtained using one-way ANOVA followed by Tukey-adjusted pairwise comparisons. Bold values indicate $p < 0.05$. In pairwise comparison labels, M-GLP-1 and M-6HIS denote the Lacto-M-GLP-1 and Lacto-M-6HIS-GLP-1 treatment groups, respectively. These analyses were exploratory and did not account for cage-level clustering because cage identifiers were unavailable for the histological subset.
